# A functionalized porous Al current collector enables high-energy density anode-free Na batteries

**DOI:** 10.1126/sciadv.adx7124

**Published:** 2025-08-20

**Authors:** Yongling An, Zhihao Pei, Deyan Luan, Xiong Wen (David) Lou

**Affiliations:** Department of Chemistry, City University of Hong Kong, 83 Tat Chee Avenue, Kowloon, Hong Kong, 999077, China.

## Abstract

Anode-free Na batteries offer the highest possible energy density but suffer from rapid capacity decay resulting mainly from the formation of Na dendrites and large volume change. Here, we design a functionalized porous Al current collector created through template-free electrodeposition and physical separation methods to modulate Na growth behavior. The controlled porous architecture, featuring interconnected ligament-channel networks, reduces structural stress and inhibits dendritic Na formation by lowering local current density and uniformizing ion flux. In addition, the abundant sodiophilic active sites boost reaction kinetics, decrease Na nucleation barrier, and subsequently manipulate homogeneous Na nucleation. Consequently, the porous Al host exhibits high reversibility of dendrite-free Na plating/stripping behavior. A proof-of-concept 4.3 V-class pouch cell achieves an energy density of up to 420.4 watt-hours per kilogram and stable cycling performance with 84.9% capacity retention over 100 cycles under anode-free conditions, providing a pathway for the design of anode-free Na batteries for practical applications.

## Teaser

Functionalized porous Al serves as a sodiophilic host for high-energy density anode-free Na batteries.

## INTRODUCTION

Rechargeable Na-metal batteries (NMBs) hold great promise for electrical energy storage benefiting from the merits of Na metal anodes, such as high theoretical capacity, low redox potential, and abundant Na resources (*1*, *2*). However, their practical application is restricted by the excessive use of Na anodes, which introduces several inherent problems in NMBs (*3*, *4*). Specifically, excessive use of Na metal compromises the energy density of NMBs (*5*, *6*). This creates a practical challenge in the field of battery technology as most materials will be wasted, further increasing production costs (*7*, *8*). Second, the NMBs with extra Na metal anodes show artificially enhanced performance because the excess Na compensates for any Na losses during electrochemical processes (*9*, *10*). However, accessing certain properties, particularly stability, may pose challenges in practical applications (*11*, *12*). Also, the sticky and soft nature of Na anode complicates the processing and molding of ultrathin metal anodes (*13*, *14*). Besides, the inadequate air stability of Na metal presents a challenge for the mass production of NMBs (*15*, *16*).

**Fig. 1.**
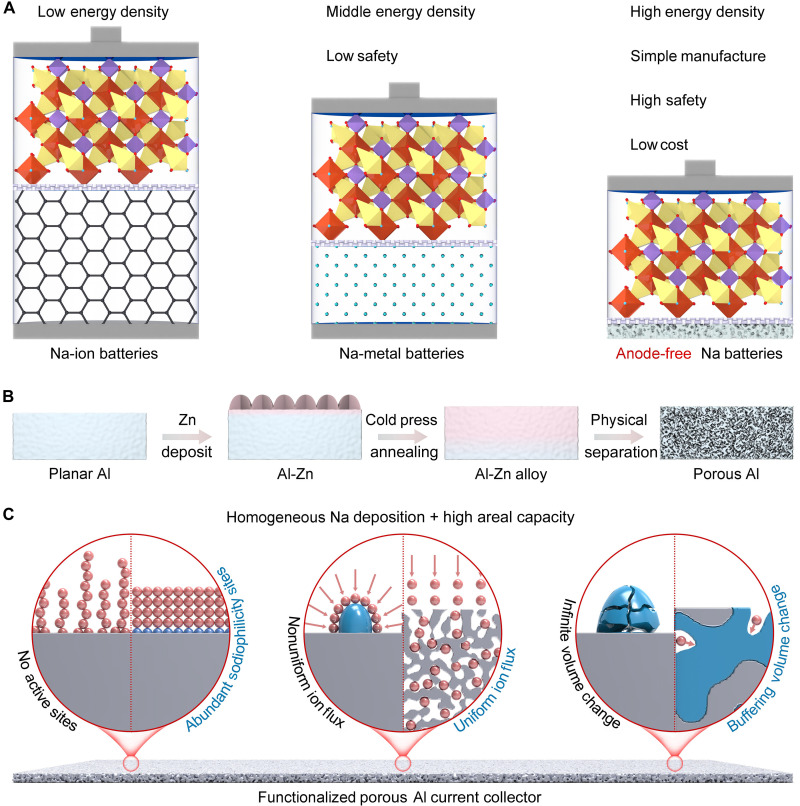
Schematic illustration. (**A**) Comparison of Na-ion batteries, NMBs, and AFNBs. (**B**) Synthesis process and (**C**) main features of porous Al.

The utilization of anode-free Na batteries (AFNBs) has the potential to overcome the aforementioned challenges (*17*–*19*). This configuration eliminates the utilization of anodes, which not only enhances the energy density of cells by reducing its volume and weight but also simplifies the production process to reduce cost (Fig. 1A) (*20*–*23*). In AFNBs, the “real anode” is created during the initial charging process (*24*, *25*). The Na generated on the anode side is consistently encapsulated without exposure to air, ensuring that no Na is lost, as active Na^+^ shuttles originate exclusively from the cathodes (*26*, *27*). However, the operation of AFNBs leads to the consumption of active Na and increases susceptibility to breakage (*28*, *29*). In addition, the nonuniform plating morphology induces the formation of “dead Na” during the electrochemical process, leading to low coulombic efficiency (CE) (*30*, *31*). These factors together with others lead to rapid capacity decay.

To tackle these challenges, many efforts have been dedicated to homogenizing Na growth (*32*, *33*). Researchers have established that the electrode current collector plays a crucial role in dendrite formation and growth (*34*–*36*). Reducing the local current density can retard the initiation of dendrite formation and reduce the growth rate (*37*, *38*). Porous frameworks have been reported to facilitate Na deposition, improving the plating area to reduce the local current density (*39*, *40*). However, in most cases, it is necessary to integrate these skeletons with the existing current collector in an additional step, thereby enhancing both polarization and electrical resistance. In addition, the complex fabrication process, along with the uncontrollable pore structure and high cost, further hinders the practical application. Moreover, effectively suppressing the formation of Na dendrites at high current densities remains a persistent challenge (*19*, *32*, *35*). Accordingly, it is crucial to design a controllable and scalable strategy for producing a three-dimensional (3D) conductive skeleton that can be effectively integrated with the current collector, which is of high significance for practical applications.

In this work, we design a functionalized porous Al current collector as a highly reversible host to modulate the growth of Na metal. The porous Al is constructed using template-free electrodeposition and physical separation techniques (Fig. 1B). The 3D conductive framework offers ample space for Na plating and improves structural stability by accommodating volume change as well as promotes compact Na growth by homogenizing ion flux and decreasing the local current density (Fig. 1C). Besides, the abundant sodiophilic active sites with low nucleation barriers uniformize Na nucleation/growth and advance reaction kinetics. Accordingly, the dendrite-free Na plating/stripping is realized using the porous Al host, exhibiting a high average CE of 100% at 10 mA cm^−2^ and 20 mAh cm^−2^ over 900 hours. A proof-of-concept 4.3 V-class anode-free pouch cell achieves an energy density of 420.4 Wh kg^−1^ (based on the active material of both anode and cathode) and demonstrates robust cycling performance with 84.9% capacity retention over 100 cycles.

## RESULTS

### Synthesis and structural characterization of porous Al

**Fig. 2.**
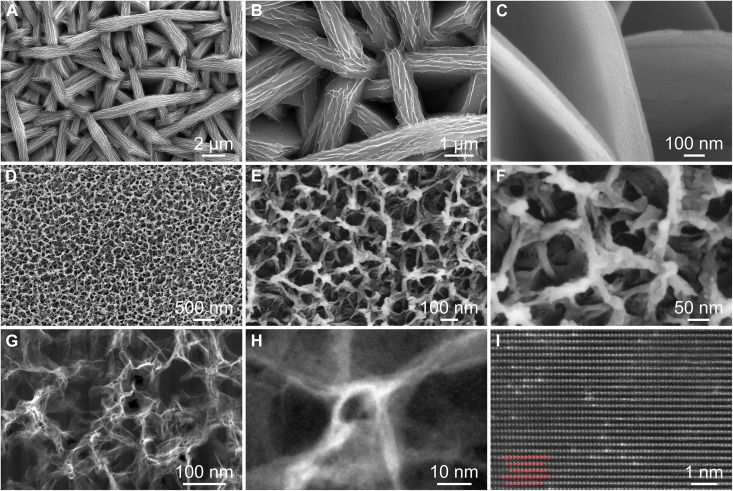
Morphology characterization of Al-Zn and porous Al. (**A** to **C**) FESEM images of Al-Zn. (**D** to **F**) FESEM, (**G** and **H**) TEM, and (**I**) high-angle annular dark-field scanning TEM images of porous Al.

Porous Al foil is constructed from commercial Al foil through template-free electrodeposition, annealing, and subsequent physical separation techniques. First, Zn metal is electrodeposited onto the planar Al surface (figs. S1 and S2). Figure 2 (A to C) and fig. S3 show field-emission scanning electron microscope (FESEM) images of the Al-Zn at various deposition current densities. At a current density of 0.2 mA cm^−2^, uniform Zn nanoflowers composed of many nanosheets are synthesized on the Al foil surface. Increasing the current density from 0.4 to 0.6 mA cm^−2^ results in the growth of Zn nanosheets to form interconnected nanospheres. As the current density reaches 0.8 mA cm^−2^, the Zn nanosheets grow into thick and uniform micrometer-sized sheets. X-ray diffraction (XRD) patterns illustrate the existence of characteristic peaks for both Zn and Al (fig. S4), demonstrating the successful deposition of Zn metal on the Al foil. Besides, as the current density increases, the intensity of the Zn characteristic peaks increases, while the intensity of the Al characteristic peaks decreases, in accordance with the energy-dispersive spectroscopy (EDS) results (fig. S5). Subsequently, the Al-Zn foil is cold-pressed and annealed to produce the Al-Zn alloy (figs. S6 and S7).

Using a physical separation approach, the porous Al foil is obtained from the Al-Zn alloy. Zn metal gradually evaporates and can be recycled during this process, making it both economical and environmentally friendly. Once the Zn metal volatilizes, the porous structure with interconnected network frameworks is formed. During this process, an Al_2_O_3_ oxide layer inevitably forms on the porous Al surface, which is conducive to stabilizing porous structures of the Al host (*41*). The porosity of the resulting porous Al varies depending on the deposition current density (figs. S8 and S9). At a current density of 0.2 mA cm^−2^, the surface becomes rough, and the porous framework gradually forms. Further increasing the current density from 0.4 to 0.6 mA cm^−2^ leads to the formation of the porous structure. When the current density increases to 0.8 mA cm^−2^, the porous structure with high porosity is obtained (Fig. 2, D to F). Thus, the porosity of the porous Al can be controlled by regulating the plating current density. This can be ascribed to the fact that the Zn metal is deposited more densely on the Al foil surface with increasing current density. As displayed in the XRD patterns and EDS spectra (figs. S10 and S11), the characteristic peaks of Al are observed, indicating the successful fabrication of porous Al (*41*). Besides, the intensity of the ZnO characteristic peaks is low, proving the presence of only a small amount of ZnO in the product. The residual ZnO can function as high-affinity Na binding sites to decrease the Na deposition energy barrier and consequently uniformize Na nucleation and growth (*42*). Transmission electron microscopy (TEM) images further disclose the porous structure of the porous Al, which contains abundant pores (Fig. 2, G and H). This structure is beneficial for homogenizing ion flux, decreasing local current density, releasing internal stress, and buffering volume changes (*39*). The lattice fringe of Al is observed (Fig. 2I), providing additional evidence of the successful synthesis of porous Al. Compared to Cu foil, Al foil, which offers an advantage in weight (fig. S12), can function as a current collector for both anodes and cathodes of Na batteries, as it does not form an alloy with metallic Na (fig. S13).

### Theoretical calculation and Na deposition behavior

**Fig. 3.**
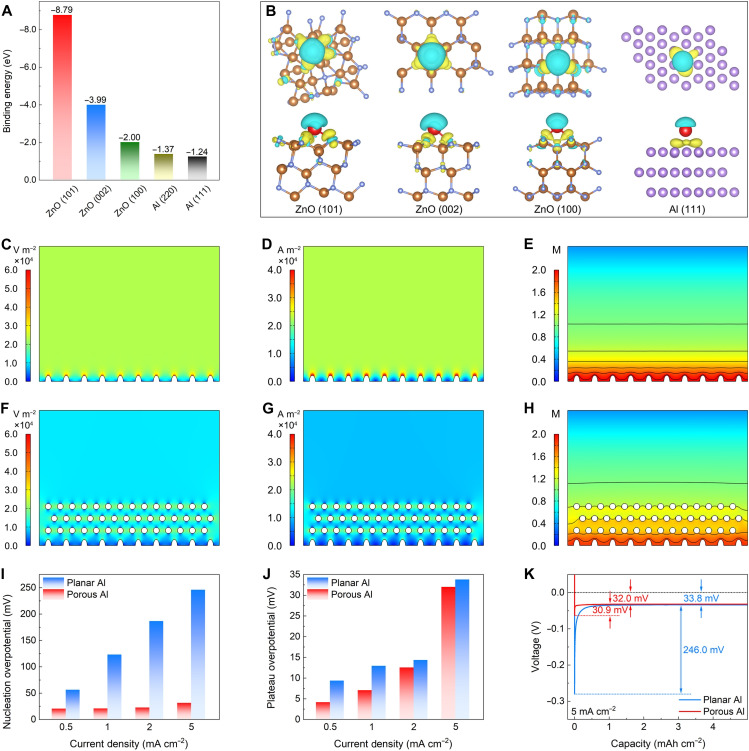
Theoretical and electrochemical results of porous Al host. (**A**) Summary of the calculated binding energy of Na atoms with Al and ZnO. (**B**) Interfacial charge-density models of Al and ZnO with Na atom adsorption. Simulated distributions of (**C** and **F**) electric field, (**D** and **G**) current density, and (**E** and **H**) electrolyte concentration on [(C) to (E)] planar Al and [(F) to (H)] porous Al. (**I**) Nucleation overpotential and (**J**) plateau overpotential of different hosts tested at different current densities of 0.5, 1.0, 2.0, and 5.0 mA cm^−2^. (**K**) Voltage-capacity profiles during Na nucleation on various hosts tested at a current density of 5.0 mA cm^−2^.

To understand Na nucleation behavior and disclose sodiophilic nature, density functional theory (DFT) calculation is carried out (figs. S14 and S15). The sodiophilicity refers to the ability to absorb and bond with Na, effectively reducing the energy barrier of Na nucleation (*43*, *44*). The binding energy between the host and Na atoms is summarized as a quantitative descriptor of sodiophilicity (Fig. 3A). Impressively, ZnO (101) shows the strongest binding energy, implying the sodiophilic nature of ZnO (*42*). Moreover, DFT-based interfacial charge density further discloses the strong interaction between Na atom and ZnO with evident charge transfer at the interface (Fig. 3B). To reveal the Na growth behavior during the electrochemical process, finite element simulations are executed to analyze the distribution of electric field intensity, current density, and electrolyte concentration on the planar Al and porous Al hosts. The nucleation and deposition of Na depend on the electric field distributions at the interface. The electric field distribution on the planar Al surface exhibits obvious variation in intensity gradient during the Na plating process (Fig. 3C). In contrast, the evenly distributed electric field on the porous Al surface ensures uniform Na deposition, resulting in a smooth and flat surface (Fig. 3F). The enhanced local electric field creates a region with higher charge, facilitating the nucleation of Na^+^ deposition. Because of the tip effect, these protuberances gradually transform into large dendrite flakes, ultimately resulting in cell failure. The results highlight the synergistic effects of the porous skeleton and sodiophilic active sites of porous Al. Besides, simulations of current density and electrolyte concentration on the planar Al and porous Al reveal nonuniform distributions on the planar Al surface, which is attributed to its uneven and defective surface (Fig. 3, D and E). This nonuniformity results in uneven Na deposition and rapid dendrite formation. In comparison, the distributions of current density and electrolyte concentration on the porous Al exhibit smaller differences (Fig. 3, G and H), resulting in dense and uniform Na plating. When used as a host for Na deposition/dissolution, the Na nucleation overpotential and plateau overpotential are investigated (fig. S16). The porous Al host shows lower nucleation overpotential and plateau overpotential compared to the planar Al host at different current densities, implying decreased Na nucleation barriers on the porous Al due to its abundant sodiophilic species (Fig. 3, I and J). Specifically, the nucleation overpotential and plateau overpotential for porous Al are 30.9 and 32.0 mV (Fig. 3K), respectively, which are lower than that of planar Al.

**Fig. 4.**
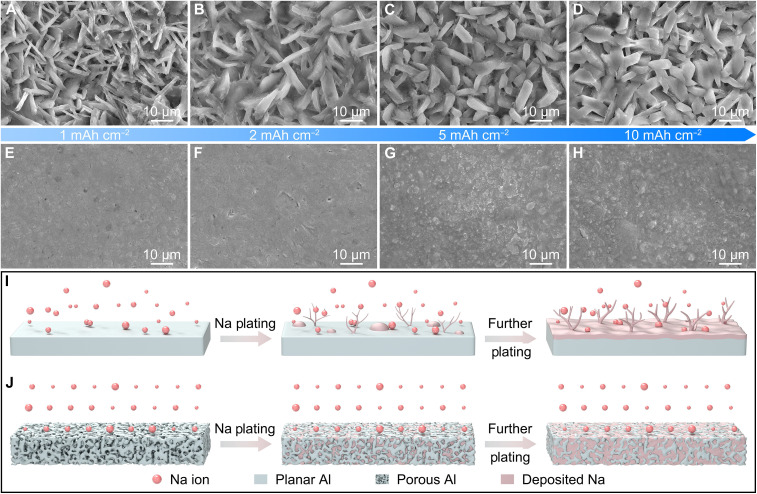
Na plating behaviors on planar Al and porous Al. FESEM images of (**A** to **D**) planar Al and (**E** to **H**) porous Al after Na deposition at a current density of 1 mA cm^−2^ with different capacities of [(A) and (E)] 1 mAh cm^−2^, [(B) and (F)] 2 mAh cm^−2^, [(C) and (G)] 5 mAh cm^−2^, and [(D) and (H)] 10 mAh cm^−2^. (**I** and **J**) Schematic illustration of Na plating on planar Al and porous Al.

To confirm the superiority of the porous Al host, the Na plating behavior on different substrates is investigated (fig. S17). At a plating capacity of 1 mAh cm^−2^, irregular and inhomogeneous Na dendrites are generated because of charge concentration resulting from the nonuniform surface of planar Al (Fig. 4A) (*26*). The initial formation of Na dendrites promotes further charge generation, exacerbating the growth of Na dendrites (*37*). As a result, increasing the plating capacity from 2 to 10 mAh cm^−2^ leads to the formation of large Na dendrites (Fig. 4, B to D), resulting in potential safety problems. In comparison, Na deposition behavior on the porous Al is clearly more uniform. At a plating capacity of 1 mAh cm^−2^, the porous skeleton is filled with deposited Na without obvious dendrite formation (Fig. 4E). With further plating, a smooth and dense surface is observed in the porous Al as the plating capacity increases from 2 to 5 mAh cm^−2^, instead of a dendritic morphology (Fig. 4, F and G). When the plating capacity of Na reaches a high value of 10 mAh cm^−2^, a relatively flat and compact surface is achieved (Fig. 4H). Even at a higher plating capacity of 20 mAh cm^−2^, the porous Al shows a dense surface without any visible Na dendrites, which differs from the dendritic morphology observed in the planar Al (figs. S18 and S19). This excellent regulation of Na deposition effectively reduces the risk of short circuit, enabling a long cycle life at high current densities and plating capacities. On the basis of these observations, the Na deposition process on planar Al and porous Al hosts is schematically illustrated. For the planar Al, Na is preferentially plated on the tip of early formed Na deposits where the electric field is concentrated, ultimately resulting in the uncontrolled Na dendrite growth and low CE (Fig. 4I). In contrast, the improved regulation of Na plating is ascribed to the unique porous framework and abundant sodiophilic species of the porous Al host (Fig. 4J). More specifically, the porous network can reduce the local current density, uniformize Na^+^ ion flux, release internal stress, and buffer volume change, thus enabling dendrite-free and high-capacity Na plating. The abundant sodiophilic species promote an ultralow nucleation barrier, facilitating homogeneous Na nucleation and growth. As a result, uniform and dense Na plating with high reversibility can be realized on the porous Al host.

### Electrochemical performance

**Fig. 5.**
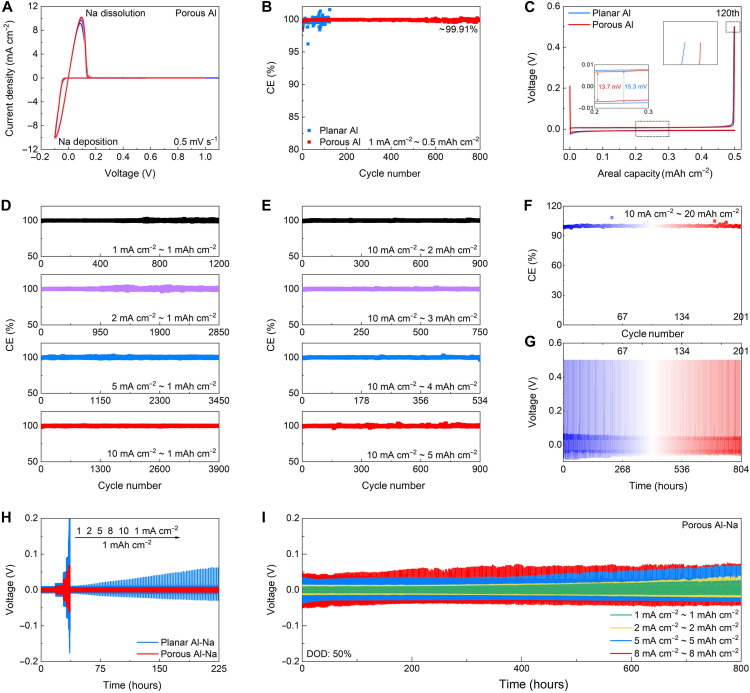
Electrochemical performance of different electrodes. (**A**) CV curves of Na//porous Al cell tested at a scan rate of 0.5 mV s^−1^. (**B**) CE plots and (**C**) corresponding plating/stripping voltage profiles at the 120th cycle of Na//planar Al and Na//porous Al cells tested at 1 mA cm^−2^ and 0.5 mAh cm^−2^. (**D** and **E**) CE plots of Na//porous Al cell tested at various (D) current densities and (E) areal capacities. (**F**) CE plots and (**G**) corresponding voltage-time profiles of Na//porous Al cell tested at 10 mA cm^−2^ and 20 mAh cm^−2^. (**H**) Rate capability of Na//planar Al-Na and Na//porous Al-Na cells tested at various current densities with a capacity of 1 mAh cm^−2^. (**I**) Cycling performance of Na//porous Al-Na cell tested at various current densities and areal capacities.

To investigate the Na deposition/dissolution efficiencies, which reveal the operational mode in anode-free designs, asymmetric cells are assembled and measured at various current densities and areal capacities. Figure 5A illustrates the cyclic voltammograms (CVs) of the Na//porous Al cell. After the first cycle, the positions and shapes of Na plating/stripping peaks remain almost unchanged compared to those of the Na//planar Al cell (fig. S20). Besides, the current density enhances remarkably, indicating improved reaction kinetics benefiting from homogeneous Na growth. The Na//planar Al cell delivers low CE values of around 99.7% and substantial CE fluctuations (Fig. 5B and fig. S21). The voltage fluctuations suggest potential failure resulting from either localized short circuiting or the reconnection of fragmented Na plats that detach from the electrode (fig. S22). For comparison, the porous Al without ZnO layer is synthesized through an electrochemical method [porous Al (E), figs. S23 and S24] (*38*). The porous Al (E) host delivers improved CE values of about 99.78% for 440 cycles (fig. S25). Besides, the Na//porous Al cell displays a long cycle life of 800 cycles (Fig. 5B) and high CE values of about 99.91% (table S1), unearthing the effectiveness of the porous design and sodiophilic ZnO species in promoting homogeneous Na growth. The porous structure is well maintained during the Na plating/stripping processes, as demonstrated by the FESEM result of porous Al after 100 cycles (fig. S26). Besides, the corresponding plating/stripping voltage profiles reveal that the Na//porous Al cell exhibits a small voltage hysteresis of 13.7 mV (Fig. 5C), which is lower than that of the Na//planar Al cell (15.3 mV), implying decreased polarization and improved reaction kinetics for the porous Al (fig. S27). The CE value of the Na//porous Al cell at the 120th cycle is higher than that of the Na//Al cell, consistent with the results shown in Fig. 5B. Besides, the porous Al hosts with different porosities show obviously improved CE values (fig. S28). In view of the uniform Zn deposition behavior, we investigate the effects of the plating capacity and current density on the CE for the Na//porous Al cell. As reported in Fig. 5D, high CE values of around 99.98, 100, 100, and 99.98% are achieved at various current densities of 1, 2, 5, and 10 mA cm^−2^, respectively, with a plating capacity of 1 mAh cm^−2^. We also evaluate the CE values at different areal capacities, ranging from 2 to 5 mAh cm^−2^ (Fig. 5E). The measured CE values are about 99.96, 99.95, 99.91, and 99.92% respectively. The plating/stripping curves of the Na//porous Al cell at different plating capacities and current densities are shown in fig. S29. We increase the Na deposition capacity to 20 mAh cm^−2^ to evaluate the stability of Na deposition (Fig. 5, F and G). High electrode reversibility (CE ≈ 100%) and stable voltage curves are observed. The performance of the Na//porous Al cells surpasses that of most previously reported NMBs with different current collectors (table S2) (*4*, *5*, *17*–*19*, *24*–*27*, *37*–*40*, *45*–*65*).

In addition, we evaluate the cycling performance of the planar Al-Na and porous Al-Na electrodes with predeposited Na under a depth-of-discharge (DOD) value of 50%. The porous Al-Na electrode shows good rate performance, with voltage hysteresis increasing to 11.6, 22.8, 57.6, 94.1, and 130.2 mV, respectively, as the current density rises from 1 to 10 mA cm^−2^ (Fig. 5H). In contrast, the planar Al-Na electrode exhibits higher voltage hysteresis and undergoes gradual voltage increases. Besides, the porous Al-Na electrode shows excellent long-term cycling performance with small voltage polarization as the current density increases from 1 to 8 mA cm^−2^, as shown in Fig. 5I and fig. S30. Even at high current density and areal capacity (10 mA cm^−2^ and 10 mAh cm^−2^), it maintains relatively small voltage hysteresis and a long life span (fig. S31 and table S3). In contrast, the planar Al-Na shows a short cycle life (fig. S32). Besides, the cycling performance of the porous Al-Na at high DOD values of 60, 70, and 80% is assessed (fig. S33). The porous Al-Na anode also delivers good durability at high DOD values. FESEM images reveal a compact and smooth surface with a dendrite-free morphology for the porous Al-Na electrode after cycling tests (fig. S34). The cycling performance of the porous Al-Na anode is comparable to that of previously reported works with different Na anodes (table S4) (*4*, *18*, *19*, *24*, *26*, *31*, *38*–*40*, *45*–*56*, *60*, *62*–*67*).

**Fig. 6.**
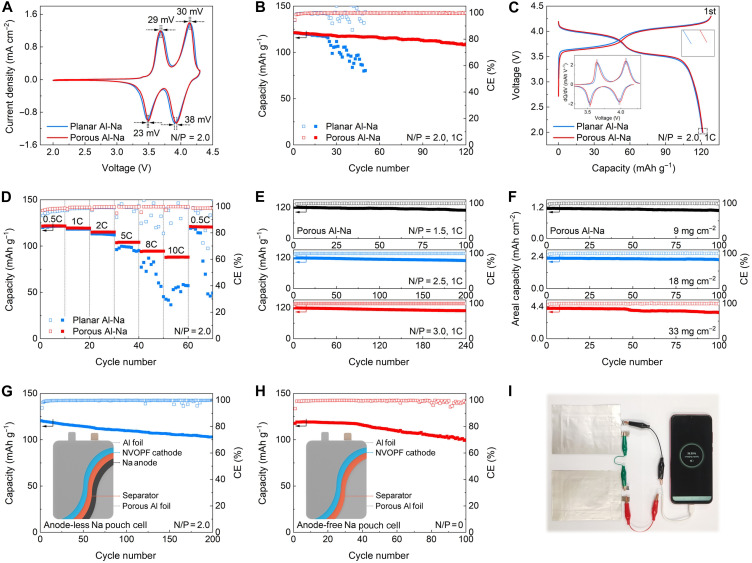
Electrochemical performance of anode-less and AFNBs. (**A**) CV profiles, (**B**) cycling performance, (**C**) galvanostatic charge/discharge voltage profiles, and (**D**) rate capability of planar Al-Na//NVOPF and porous Al-Na//NVOPF cells (N/P = 2.0). Cycling performance of porous Al-Na//NVOPF cell with (**E**) different N/P ratios and (**F**) different mass loadings of NVOPF cathode. Cycling performance of (**G**) anode-less porous Al-Na//NVOPF pouch cell (N/P = 2.0) and (**H**) anode-free porous Al//NVOPF pouch cell (N/P = 0). (**I**) Optical image of a fully charged anode-free porous Al//NVOPF pouch cell used to light up a phone.

To further demonstrate the feasibility of porous Al for practical applications, anode-less and anode-free full cells using a Na_3_V_2_O_2_(PO_4_)_2_F (NVOPF) cathode are assembled (figs. S35 and S36). The electrochemical performance of the Na//NVOPF cell is evaluated (figs. S37 and S38). The porous Al-Na//NVOPF cell displays smaller voltage polarization and higher current density than the planar Al-Na//NVOPF cell in the CV curves, demonstrating the rapid reaction kinetics facilitated by porous Al (Fig. 6A). Besides, the subsequent CV profiles display similar shapes after the first cycle, indicating a reversible electrochemical reaction (fig. S39). The porous Al-Na//NVOPF cell also delivers favorable cycling performance with 89.8% capacity retention after 120 cycles (Fig. 6B). In comparison, the planar Al-Na//NVOPF cell retains only 66.4% of its initial capacity (fig. S40), highlighting the superiority of the porous Al-Na anode. The improved performance is attributed to the inhibition of Na dendrites for the porous Al-Na (fig. S41). Figure 6C investigates the galvanostatic charge/discharge voltage profiles of the planar Al-Na//NVOPF and porous Al-Na//NVOPF cells. The porous Al-Na//NVOPF cell shows low voltage polarization and high capacity, consistent with the CV results. Moreover, the porous Al-Na//NVOPF cell delivers higher capacity than the planar Al-Na//NVOPF cell across various current densities (Fig. 6D), proving the improved rate capability of the porous Al-Na//NVOPF cell, which is verified by the galvanostatic charge/discharge voltage curves (fig. S42). The electrochemical impedance spectroscopy results exhibit a low charge transfer resistance in the porous Al-Na//NVOPF cell (fig. S43), which could account for the enhanced rate performance. At a higher current density of 2C, the porous Al-Na//NVOPF cell exhibits stable cycling performance with 93.9% capacity retention over 300 cycles (fig. S44). Besides, we investigate the effect of the negative to positive electrode capacity (N/P) ratio on the cycling performance of the porous Al-Na//NVOPF cell. Even at a low N/P ratio of 1.5, the cell still shows good cycling performance (Fig. 6E and fig. S45). As the N/P ratio increases from 2.5 to 6.0, the cycling performance is significantly improved (fig. S46). A high N/P ratio can improve cycling performance, but it also decreases the energy density of the battery. Thus, balancing this relationship is crucial in anode-less Na batteries. Besides, we compare the cycling performance of the porous Al-Na//NVOPF cell with different mass loadings of the NVOPF cathode (Fig. 6F and figs. S47 and S48). Even at a high mass loading of 33 mg cm^−2^, the cell achieves 86.2% capacity retention after 100 cycles, which outperforms previously reported anode-less Na batteries (table S5) (*27*, *45*, *47*, *50*, *56*, *60*). Besides, a pouch cell is assembled to probe the commercialization potential. As a proof of concept, an anode-less porous Al-Na//NVOPF pouch cell (N/P = 2.0) delivers a high initial CE of 94.1% and a capacity retention of 85.3% over 200 cycles (Fig. 6G and fig. S49). The anode-free configuration maximizes the energy density of the cell by reducing its volume and weight. Therefore, we also assemble an anode-free porous Al//NVOPF pouch cell (N/P = 0) to investigate its electrochemical performance (Fig. 6H and fig. S50). The anode-free pouch cell delivers a high initial CE of 93.6% and a high capacity of 99.9 mAh g^−1^ with a capacity retention of 84.9% after 100 cycles. The performance of this anode-free pouch cell outperforms the state-of-the-art AFNBs (table S6) (*5*, *18*, *19*, *24*–*26*, *31*, *34*, *37*–*40*, *45*, *47*, *50*, *55*–*57*, *59*, *61*, *62*, *66*–*68*). Besides, the anode-free porous Al//NVOPF pouch cell also shows good rate capability (fig. S51). Moreover, the electrochemical performance of the anode-free porous Al//NVOPF pouch cell tested at 0° and 40°C is evaluated (fig. S52). This anode-free pouch cell delivers stable cycling performance at both high and low temperatures. A series of visual exhibition experiments are conducted to demonstrate the practical prospect of 4.3 V-class anode-free pouch cell (Fig. 6I and fig. S53). In addition, the potential application of porous Al hosts in other AFNBs is probed. The commercial NaNi_1/3_Fe_1/3_Mn_1/3_O_2_ (NFM) and Na_4_Fe_3_(PO_4_)_2_(P_2_O_7_) (NFPP) are used as cathodes (figs. S54 and S55). The electrochemical performance of the Na//NFM and Na//NFPP cells is assessed (figs. S56 to S58). As shown in figs. S59 and S60, the anode-free porous Al//NFM and porous Al//NFPP pouch cells also exhibit stable cycling performance. These impressive results highlight the promise of porous Al current collectors in the development of high-energy density batteries.

## DISCUSSION

In summary, we have designed a functionalized porous Al as a 3D sodiophilic host for robust AFNBs. The porous framework can accommodate volume variation thus releasing structural stress and suppress Na dendrite growth by decreasing the local current density and uniformizing ion flux. Furthermore, the sodiophilic sites promote reaction kinetics, reduce Na nucleation barrier, and subsequently regulate homogeneous Na nucleation/growth. Benefiting from the multiple synergistic effects of the porous Al current collector, the Na plating/stripping reversibility is greatly enhanced with high average CE of 100% at 10 mA cm^−2^ and 20 mAh cm^−2^. As a result, the anode-less Na pouch cell (N/P = 2.0) delivers stable cycling performance with 85.3% capacity retention over 200 cycles. Besides, a proof-of-concept 4.3 V-class anode-free pouch cell (N/P = 0) shows good cycling stability of 84.9% capacity retention for 100 cycles and high energy density of 420.4 Wh kg^−1^. This work offers some anode-free prototyping to advance energy-dense batteries.

## MATERIALS AND METHODS

### Synthesis of Al-Zn foil

Al-Zn was synthesized using a template-free electrodeposition method. An Al foil was first treated with ethanol and deionized water. The treated foil was then placed in an electrode holder to serve as the working electrode. A solution was prepared by dissolving 172 mg of ZnSO_4_·7H_2_O, 13 mg of (NH_4_)_2_SO_4_, and 4 mg of cetyltrimethylammonium bromide in 100 ml of deionized water to serve as the electrodeposition solution. Zn foil was used as both the reference electrode and the counter electrode. Electrodeposition was executed to plate Zn metal at different deposition current densities. The resulting Al-Zn foil was subsequently washed with deionized water for further use.

### Synthesis of Al-Zn alloy

The as-prepared Al-Zn foil was first cold-pressed for 48 hours. Afterward, the foil was annealed in an Ar atmosphere at 350°C for 24 hours. After cooling to ambient temperature, the Al-Zn alloy foil was continuously cold-pressed for 12 hours. This process was repeated three times.

### Synthesis of porous Al

The porous Al foil was fabricated from Al-Zn alloy foil using a physical separation method. Specifically, the as-prepared Al-Zn alloy was heated under dynamic high vacuum conditions at 450°C for 5 hours. During this process, the Zn metal was evaporated at high temperature, resulting in the formation of a porous structure. In the product, a small amount of ZnO was obtained.

### Synthesis of porous Al (E)

The porous Al (E) foil without ZnO layer was synthesized from the treated Al foil using an electrochemical method. Specifically, the treated Al foil was etched in a 6% HCl solution at a current density of 1 mA cm^−2^ for 3 min.

### Materials characterizations

The phase composition was analyzed using XRD (Rigaku D/Max-KA). The structure and morphology were examined with a FESEM (JSM-7800F) and a transmission electron microscope (JEOL, JEM-1400). Elemental mapping images were characterized using a transmission electron microscope (JEOL, JEM-2100F) equipped with energy-dispersive x-ray spectroscopy. The porous Al foil was thinned using a focused ion beam–scanning electron microscope (Thermo Scientific Helios 5 UC DualBeam).

### Electrochemical measurements

Electrochemical tests were conducted using coin-type cells (CR2032). Galvanostatic charge/discharge measurements were carried out with a Neware battery test system (CT-4008-5 V 10 mA-164). CV was executed on an electrochemical workstation (CHI 660E). Electrochemical impedance spectra were measured from 100 kHz to 10 MHz (CHI 660E). A total of 1 M sodium hexafluorophosphate (NaPF_6_) in diglyme was used as the electrolyte. Glass fiber was used as the separator. In the Na plating/stripping CE measurements, Na metal was used as both the reference electrode and the counter electrode. Planar Al or porous Al foils were used as the working electrode. The Al//Na cells were first cycled for 10 cycles between 0.01 and 0.5 V at 0.05 mA for aging and activation. Subsequently, the cells were discharged (plating process) at various current densities and time and charged (stripping process) to 0.5 V. $\text{CE}=\frac{\text{stripping capacity}}{\text{plating capacity}}\times 100\%$ , which quantifies the reversibility of Na metal on the current collectors. For the cycling performance tests, Na was preplated on the planar Al and porous Al to obtain the planar Al-Na and porous Al-Na electrodes. In the anode-less and anode-free cell tests, commercial NVOPF was used as the cathode. For the preparation of cathode, NVOPF powder, Ketjen black, and polyvinylidene difluoride were mixed in a weight ratio of 7:2:1 with *N*-methyl-2-pyrrolidone solvent. Afterward, the resulting slurry was pasted onto an Al foil and dried at 150°C for 12 hours under vacuum conditions. An anode-less full cell was assembled using NVOPF as the cathode and planar Al-Na or porous Al-Na as the anode. An anode-free full cell was assembled using NVOPF as the cathode and planar Al or porous Al as the anodic current collector.
