# Supplementary material for: A functionalized porous Al current collector enables high-energy density anode-free Na batteries
Source: Sci Adv. 2025 Aug 20;11(34):eadx7124. doi: 10.1126/sciadv.adx7124 (PMC12366698; doi:10.1126/sciadv.adx7124)
Supplement: Supplementary file 1 — Supplementary Text Figs. S1 to S60 Tables S1 to S6 [file sciadv.adx7124_sm.pdf]

Science Advances
AAAS

Supplementary Materials for

A functionalized porous Al current collector enables high-energy density anode-free Na batteries

Yongling An *et al.*

Corresponding author: Xiong Wen (David) Lou, david.lou@cityu.edu.hk

Sci. Adv. **11**, eadx7124 (2025)
DOI: 10.1126/sciadv.adx7124

This PDF file includes:

Supplementary Text
Figs. S1 to S60
Tables S1 to S6

1. Supplementary Text

Computational details

The Vienna ab initio simulation package was utilized for density functional theory calculations. The interactions between ions and electrons were modeled using the projector augmented wave method. The generalized gradient approximation based on the Perdew-Burke-Ernzerhof scheme was employed for the description of electron exchange and correlation interactions. Plane-wave basis with wave function cut-off energy of 450 eV was utilized. The atomic relaxation was terminated once the total energy tolerance converged to 10^{-5} eV and the changes of the force on atoms were less than 0.02 eV $Å^{-1}$. Additionally, all structures were optimized using a (3×3×1) Monkhorst-Pack K-point grid considering the symmetry of supercell, accuracy of calculation, and cost of used time. A vacuum space of 15 Å was placed along the Z axis to avoid the interaction among the slab.

2. Figure in the Supplementary Materials

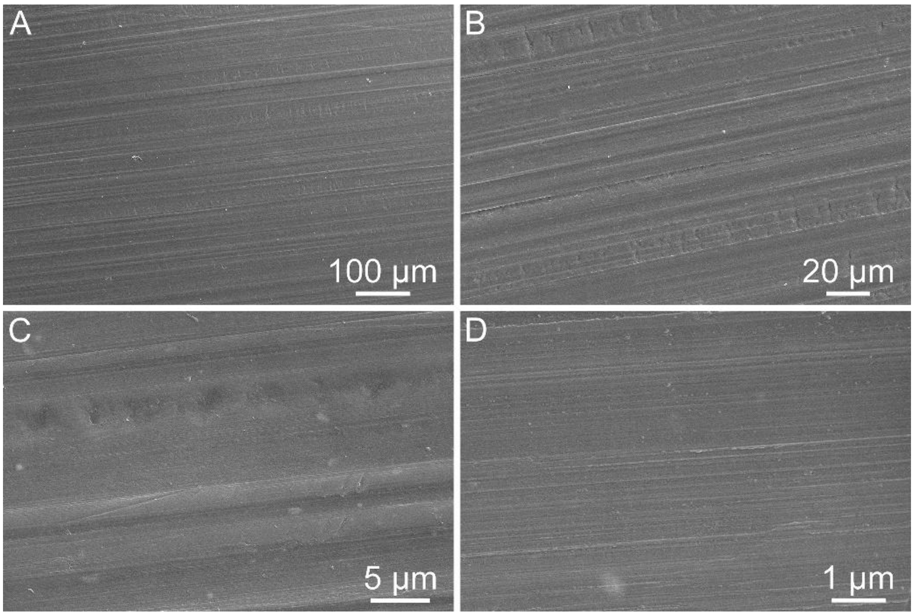

Fig. S1. FESEM measurement. FESEM images of planar Al.

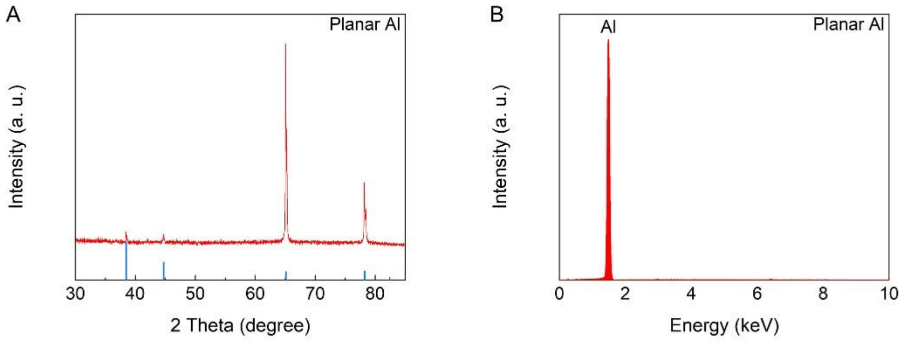

Fig. S2. XRD and EDX measurements. (A) XRD pattern and (B) EDX spectrum of planar Al.

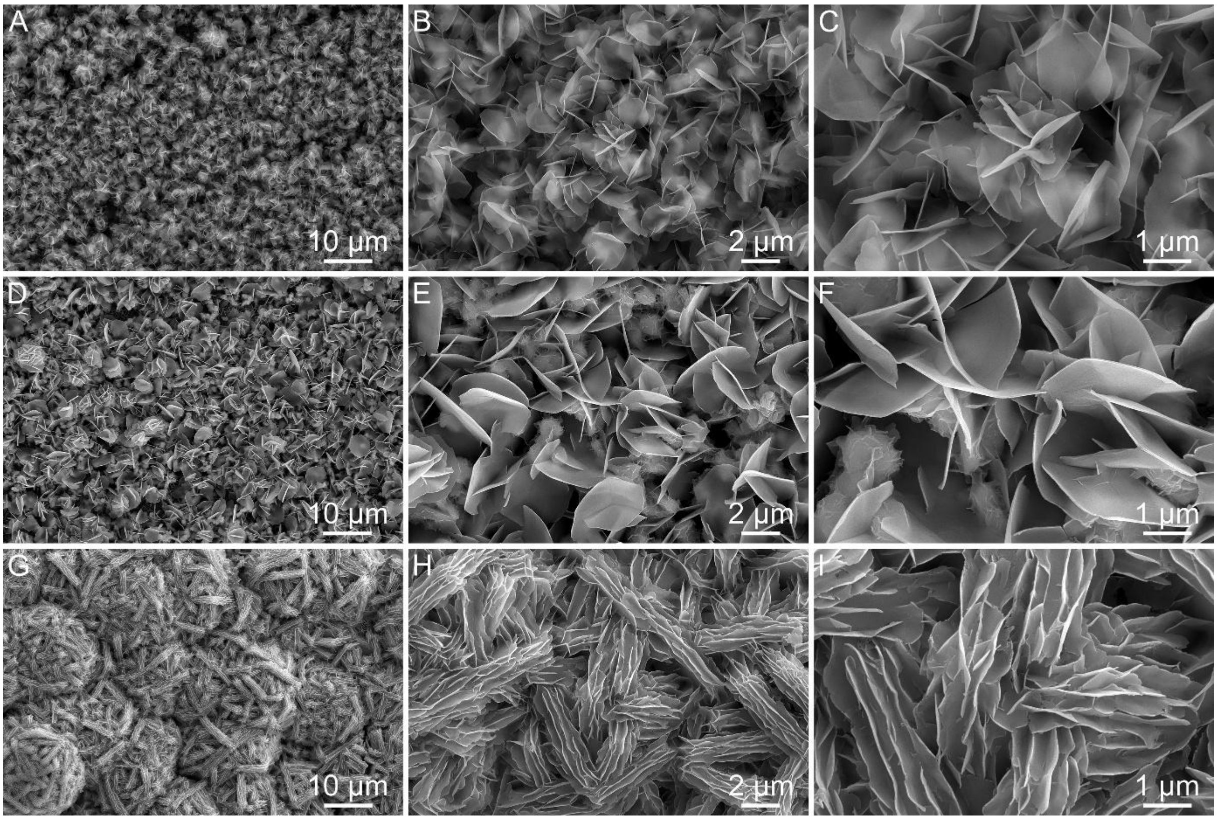

Fig. S3. FESEM measurement. FESEM images of Al-Zn with different deposited current densities. (A to C) 0.2 mA cm^{-2}, (D to F) 0.4 mA cm^{-2}, and (G to I) 0.6 mA cm^{-2}.

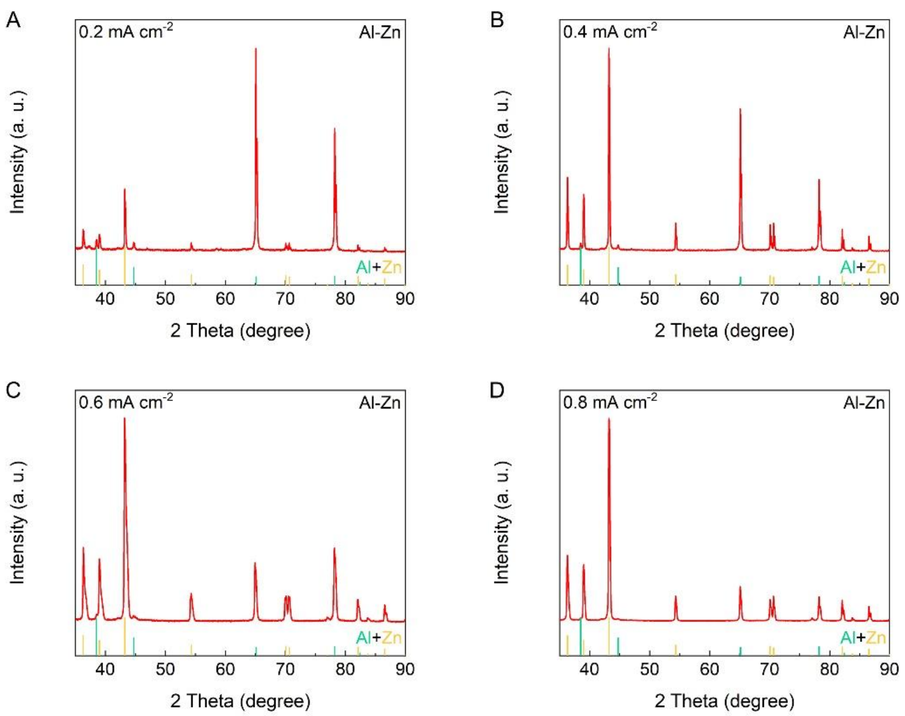

Fig. S4. XRD measurement. XRD patterns of Al-Zn with different deposited current densities. (A) 0.2 mA cm^{-2}, (B) 0.4 mA cm^{-2}, (C) 0.6 mA cm^{-2}, and (D) 0.8 mA cm^{-2}.

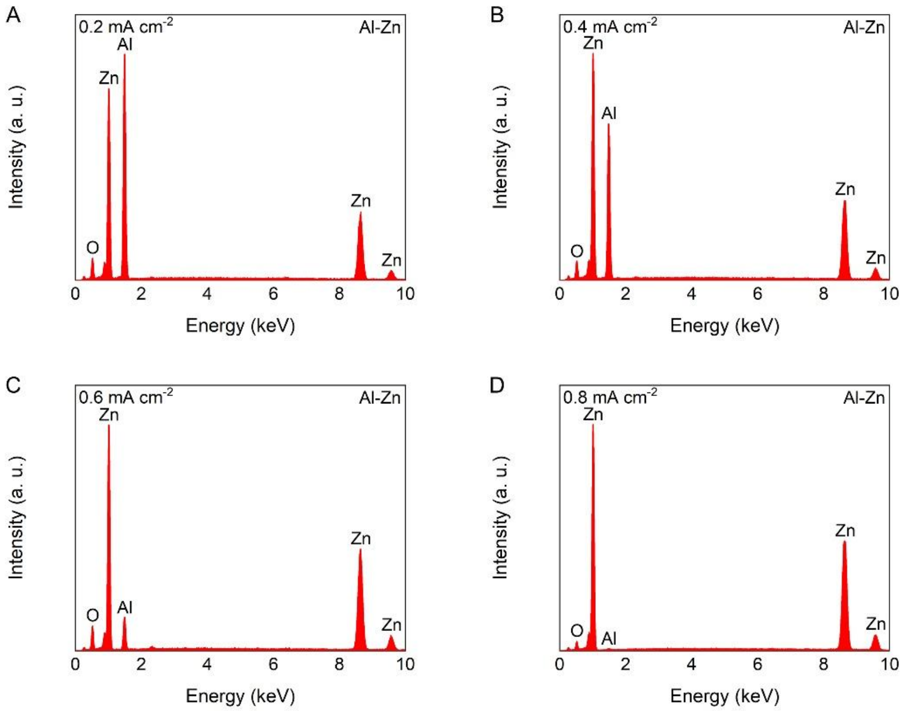

Fig. S5. EDX measurement. EDX spectra of Al-Zn with different deposited current densities. (A) 0.2 mA cm^{-2}, (B) 0.4 mA cm^{-2}, (C) 0.6 mA cm^{-2}, and (D) 0.8 mA cm^{-2}.

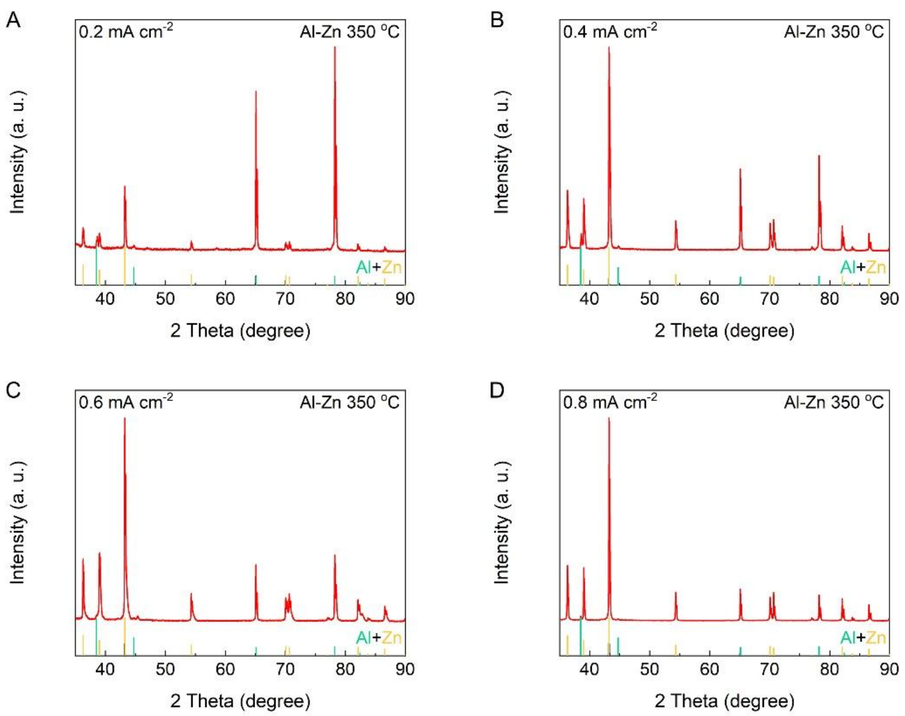

Fig. S6. XRD measurement. XRD patterns of annealed Al-Zn with different deposited current densities. (A) 0.2 mA cm^{-2}, (B) 0.4 mA cm^{-2}, (C) 0.6 mA cm^{-2}, and (D) 0.8 mA cm^{-2}.

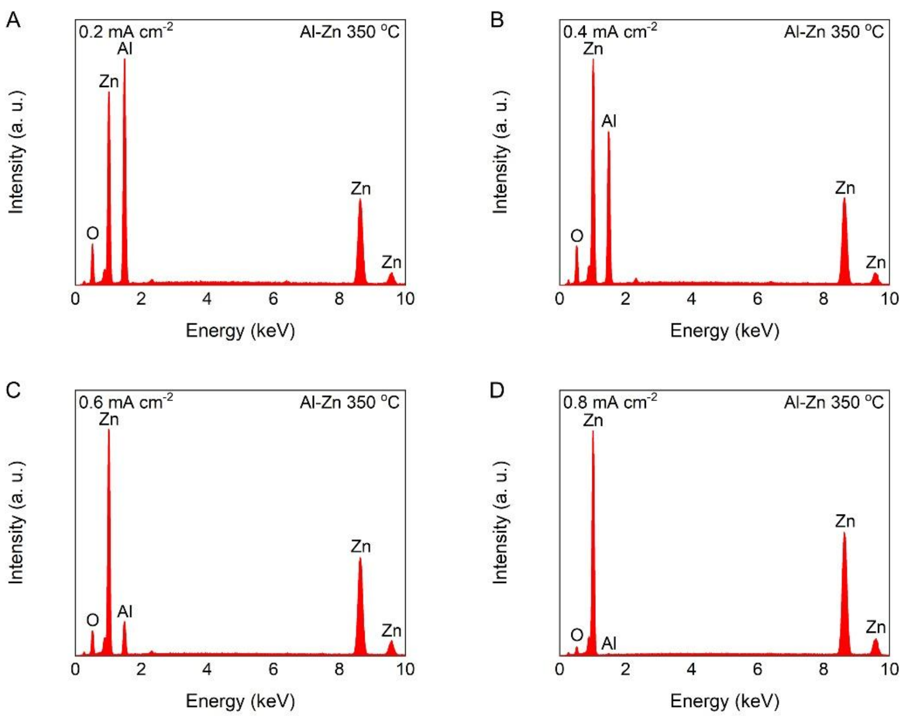

Fig. S7. EDX measurement. EDX spectra of annealed Al-Zn with different deposited current densities. (A) 0.2 mA cm^{-2}, (B) 0.4 mA cm^{-2}, (C) 0.6 mA cm^{-2}, and (D) 0.8 mA cm^{-2}.

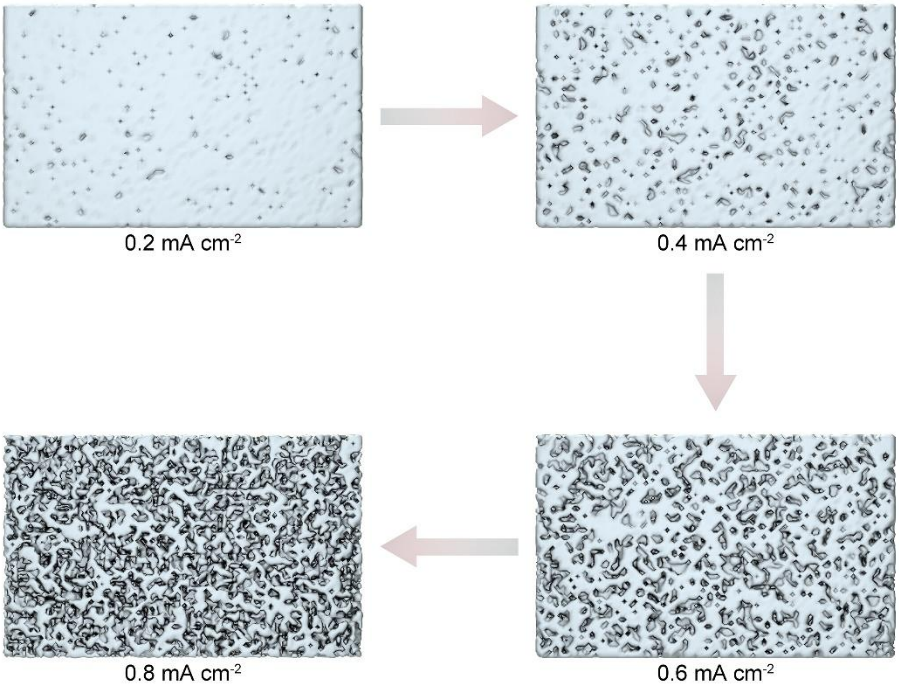

Fig. S8. Schematic illustration. Schematic illustration of porous Al with different porosities.

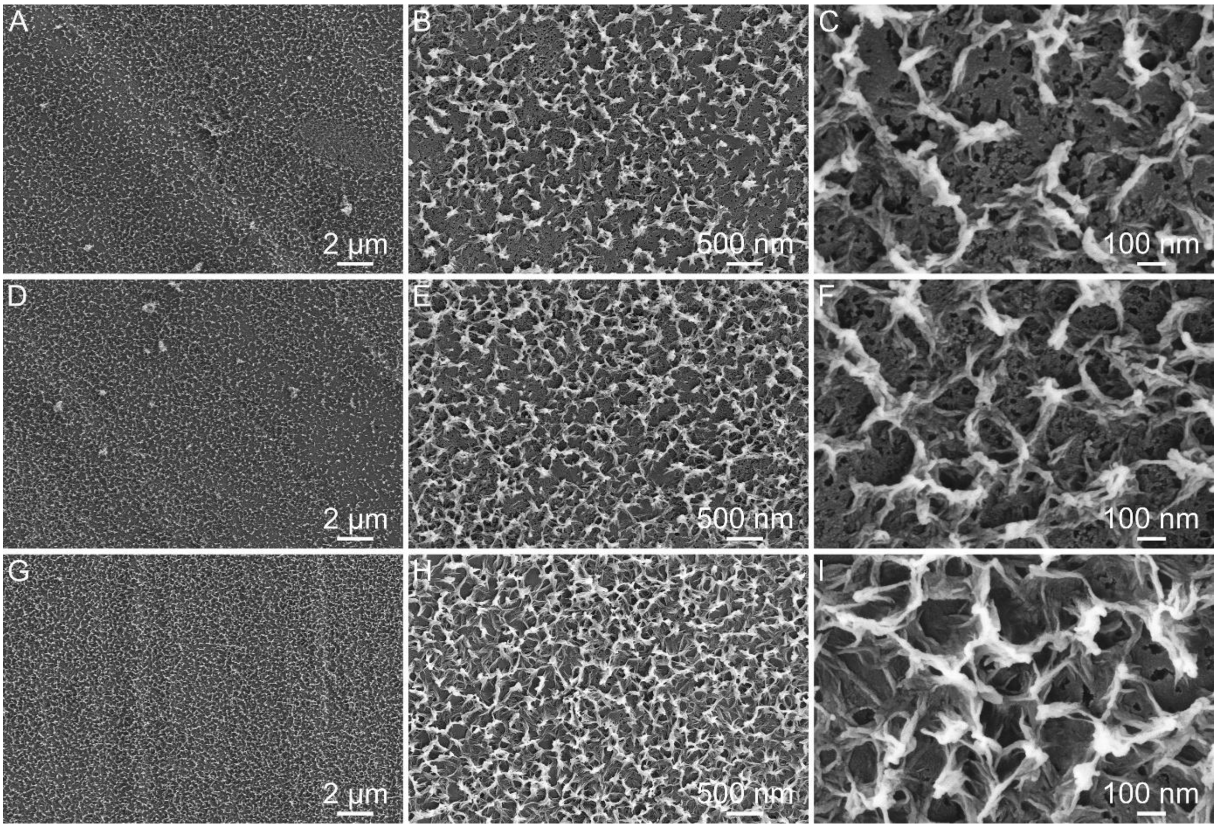

Fig. S9. FESEM measurement. FESEM images of porous Al with different deposited current densities. (A to C) 0.2 mA cm^{-2}, (D to F) 0.4 mA cm^{-2}, and (G to I) 0.6 mA cm^{-2}.

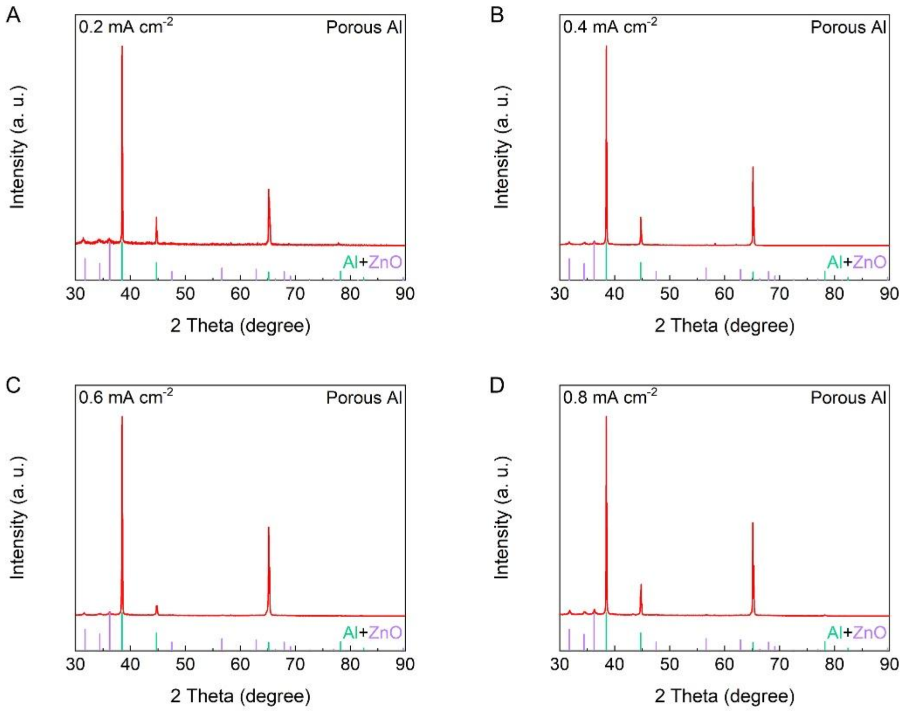

Fig. S10. XRD measurement. XRD patterns of porous Al with different deposited current densities. (A) 0.2 mA cm^{-2}, (B) 0.4 mA cm^{-2}, (C) 0.6 mA cm^{-2}, and (D) 0.8 mA cm^{-2}.

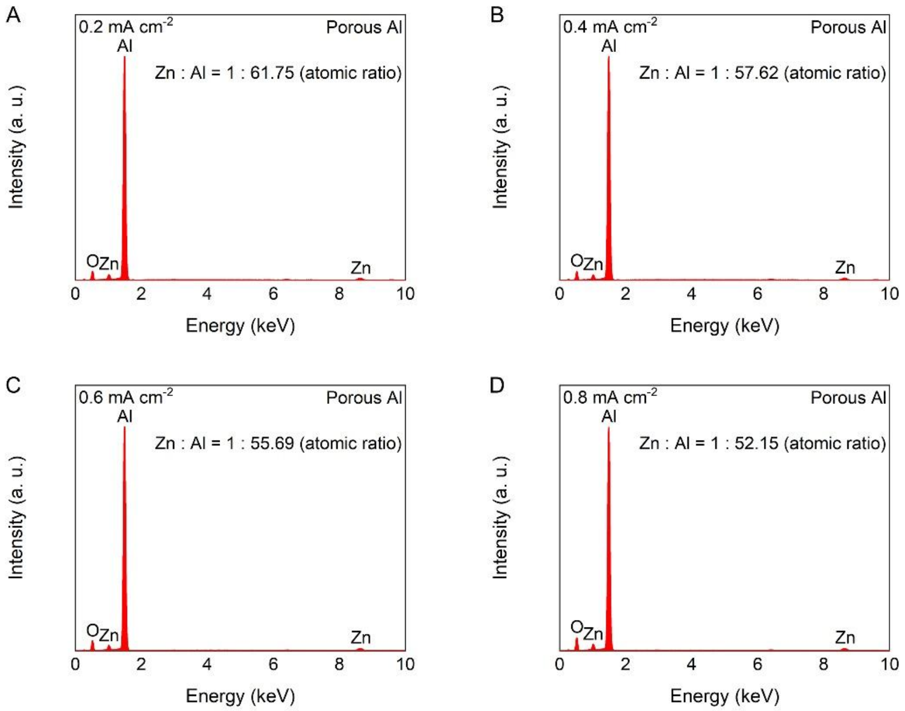

Fig. S11. EDX measurement. EDX spectra of porous Al with different deposition current densities. (A) 0.2 mA cm^{-2}, (B) 0.4 mA cm^{-2}, (C) 0.6 mA cm^{-2}, and (D) 0.8 mA cm^{-2}.

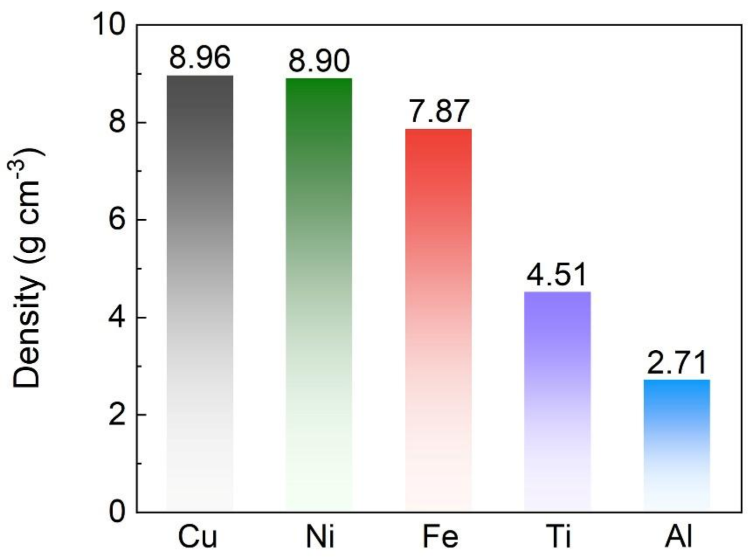

Fig. S12. Density analysis. Density of several current collectors. The thickness and areal density of Al are approximately 12 μm and 3.25 mg cm^{-2}, respectively.

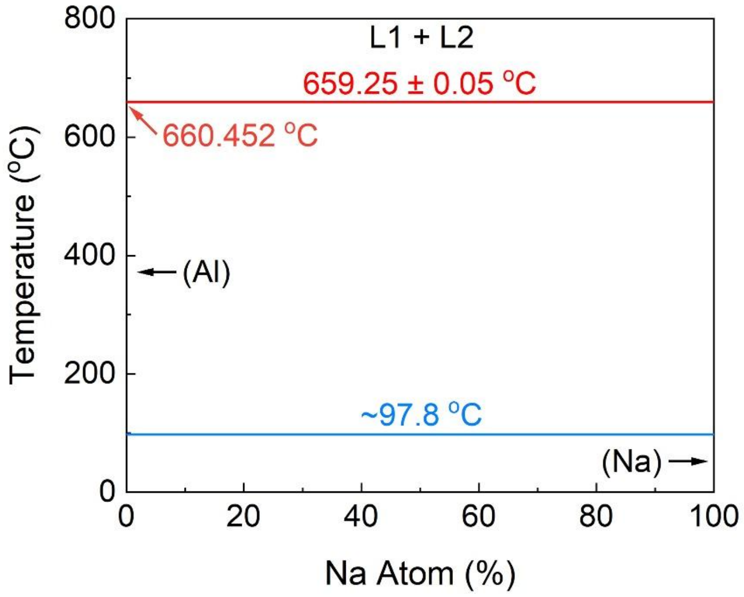

Fig. S13. Phase diagram analysis. Phase diagram of Na-Al alloy.

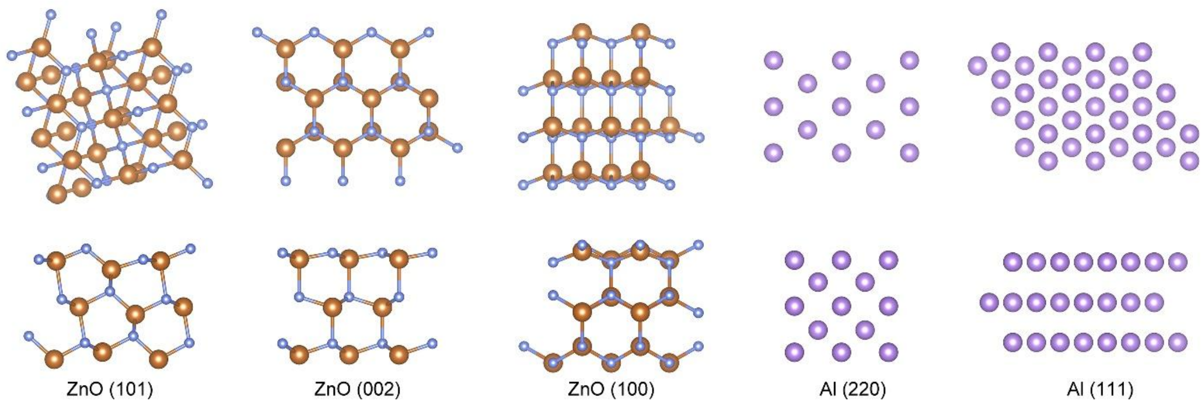

Fig. S14. Structural model. Models of ZnO (101), ZnO (002), ZnO (100), Al (220), and Al (111).

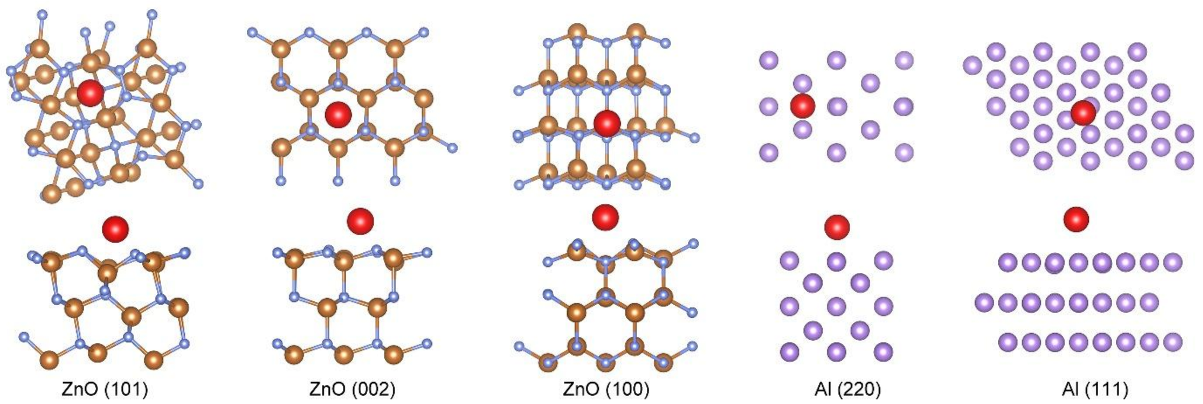

Fig. S15. Structural model. Models of ZnO (101), ZnO (002), ZnO (100), Al (220), and Al (111) after Na atom adsorption.

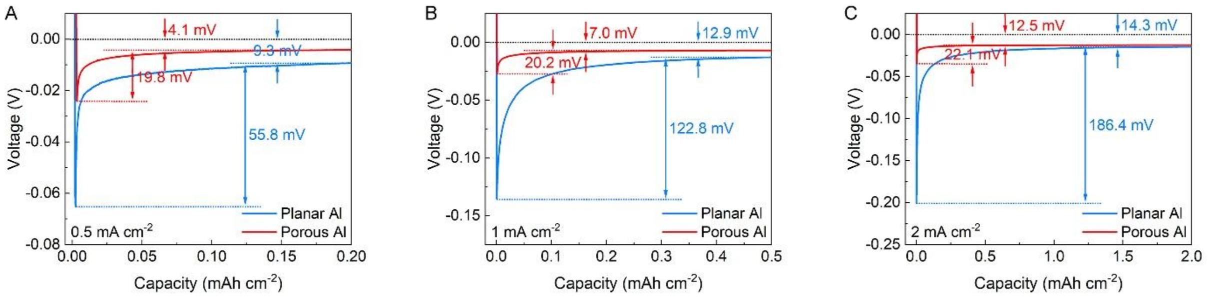

Fig. S16. Voltage-capacity analysis. Voltage-capacity curves during Na nucleation on different hosts at different current densities of (A) 0.5 mA cm^{-2}, (B) 1 mA cm^{-2}, and (C) 2 mA cm^{-2}.

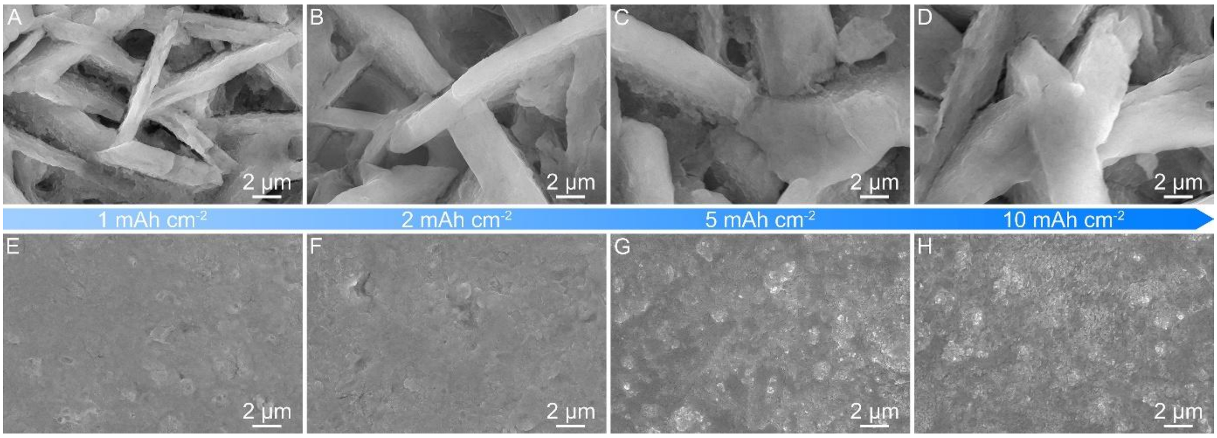

Fig. S17. FESEM measurement. Na metal deposition behavior on different current collectors. FESEM images of (A to D) planar Al and (E to H) porous Al hosts after Na plating at a current density of 1 mA cm^{-2} with different capacities of (A and E) 1 mAh cm^{-2}, (B and F) 2 mAh cm^{-2}, (C and G) 5 mAh cm^{-2}, and (D and H) 10 mAh cm^{-2}.

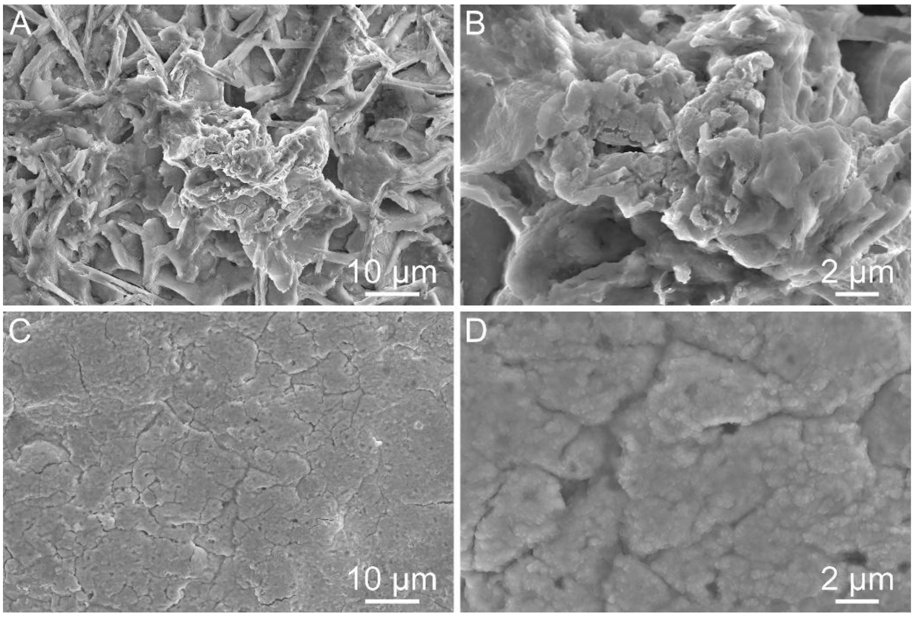

Fig. S18. FESEM measurement. FESEM images of (A and B) planar Al and (C and D) porous Al hosts after Na plating at a current density of 10 mA cm^{-2} with a capacity of 20 mAh cm^{-2}.

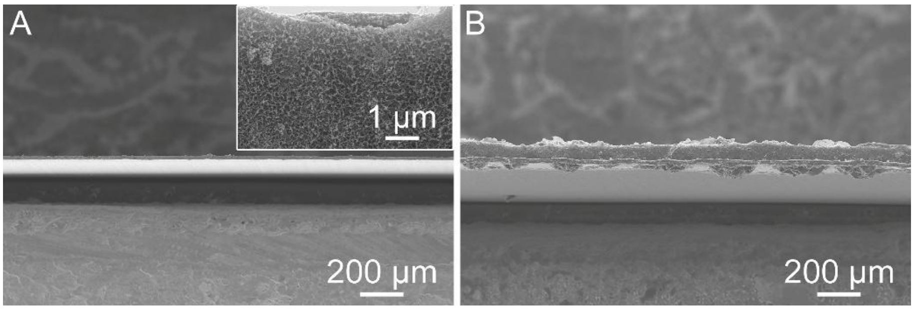

Fig. S19. FESEM measurement. Cross-sectional FESEM images of porous Al host (A) before and (B) after Na plating at a current density of 10 mA cm^{-2} with a capacity of 20 mAh cm^{-2}.

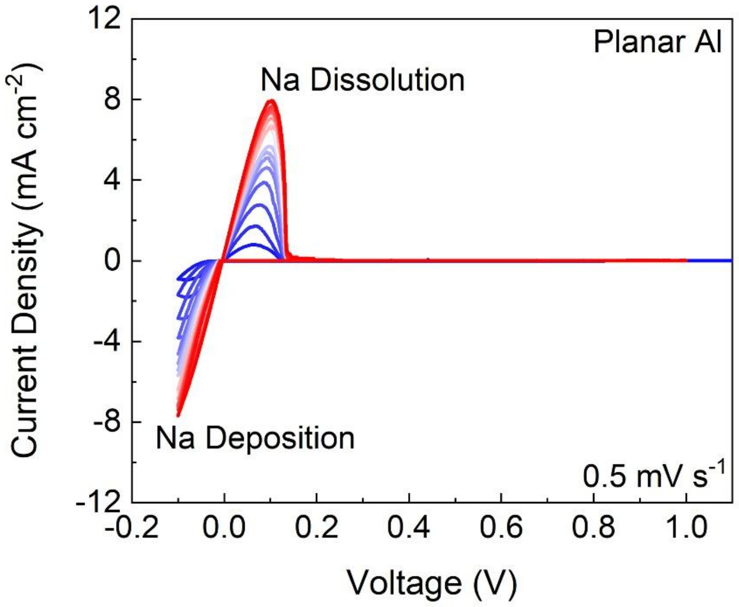

Fig. S20. CV analysis. CV curves of Na//planar Al cell at a scan rate of 0.5 mV s^{-1}.

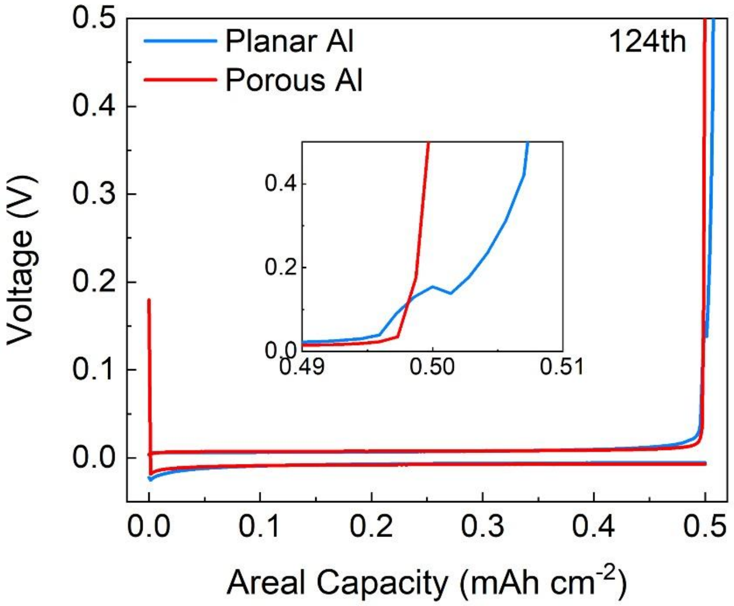

Fig. S21. CE analysis. Plating/stripping voltage profiles of Na//planar Al and Na//porous Al cells at the 124th cycle.

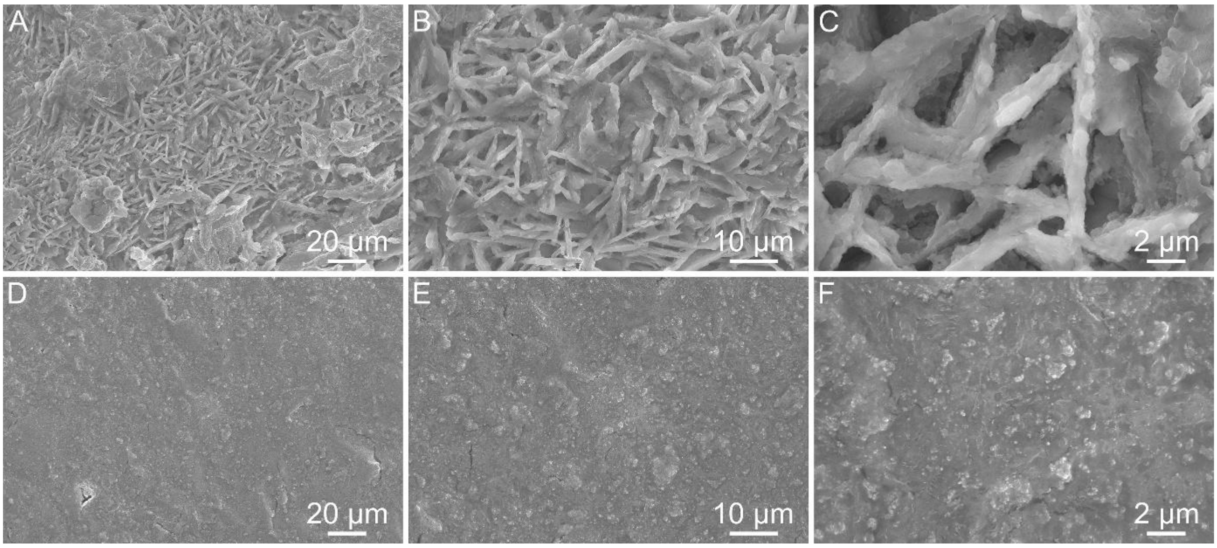

Fig. S22. FESEM measurement. FESEM images of (A to C) planar Al and (D to F) porous Al hosts after cycling.

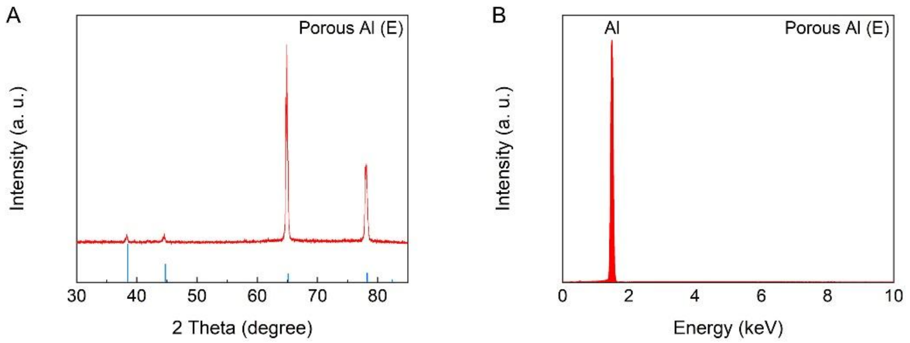

Fig. S23. XRD and EDX measurements. (A) XRD pattern and (B) EDX spectrum of porous Al (E).

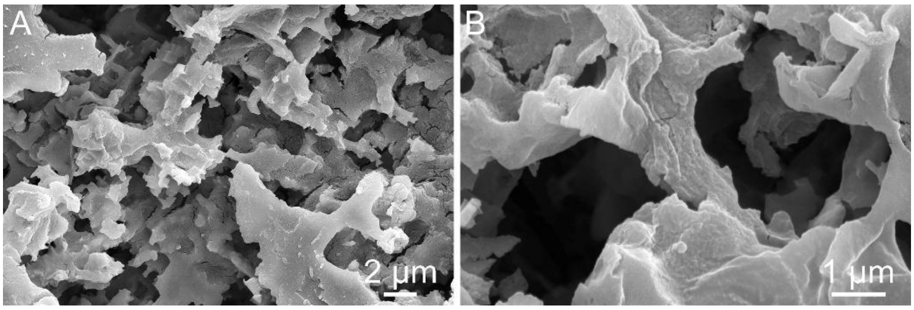

Fig. S24. FESEM measurement. FESEM images of porous Al (E).

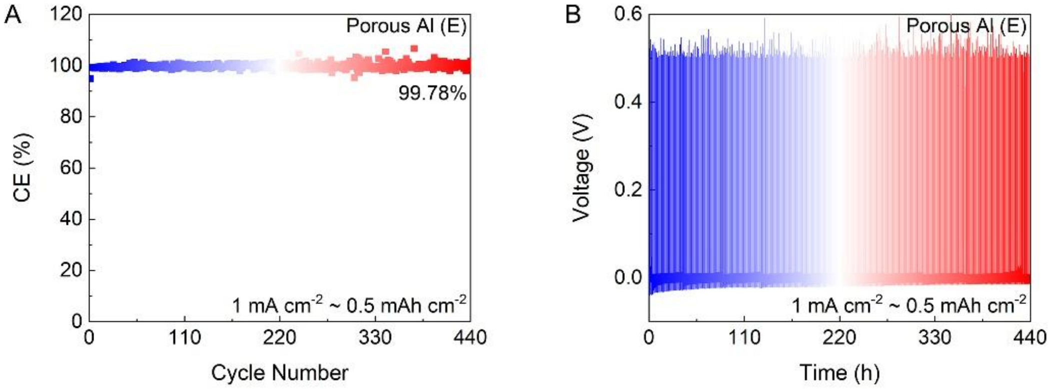

Fig. S25. CE analysis. (A) CE plot and (B) corresponding plating/stripping voltage profiles of Na//porous Al (E) cell tested at 1 mA cm^{-2} and 0.5 mAh cm^{-2}.

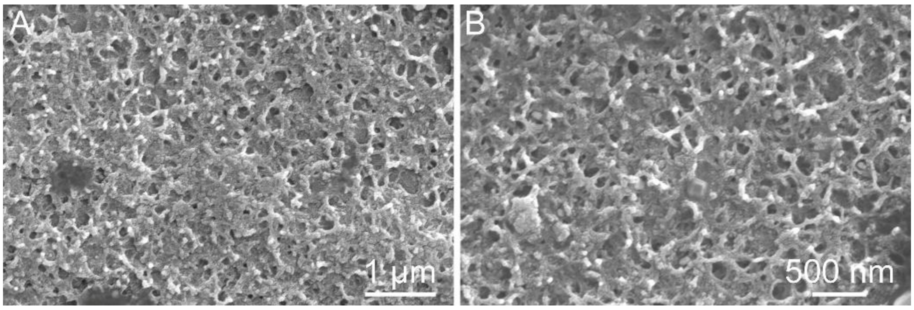

Fig. S26. FESEM measurement. FESEM images of porous Al host after 100 cycles.

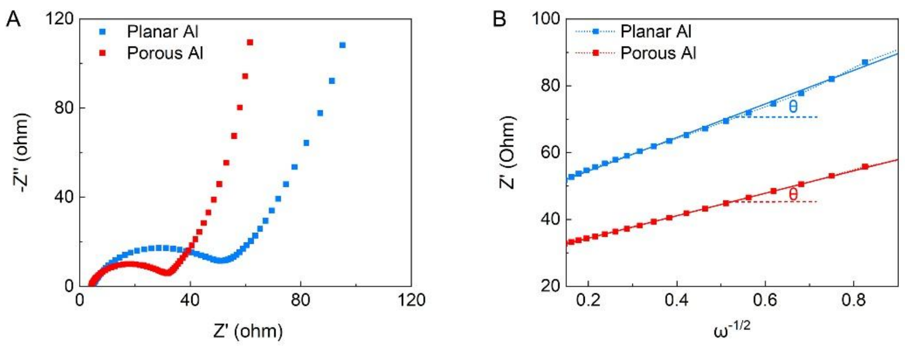

Fig. S27. EIS analysis. (A) Nyquist plots and (B) relationship plots between *Z*' and $\omega^{-1/2}$ of Na//planar Al and Na//porous Al cells after cycling.

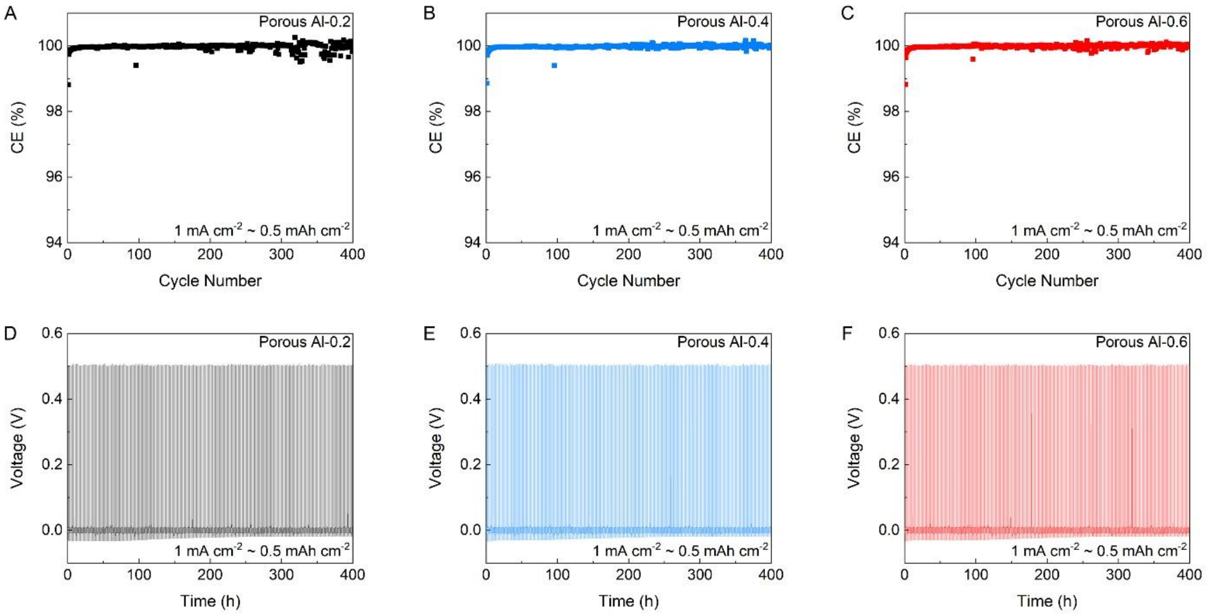

Fig. S28. CE analysis. (A to C) CE plots and (D to F) corresponding plating/stripping voltage profiles of Na//Al cells with porous Al hosts of different porosities. (A and D) porous Al-0.2, (B and E) porous Al-0.4, (C and F) porous Al-0.6.

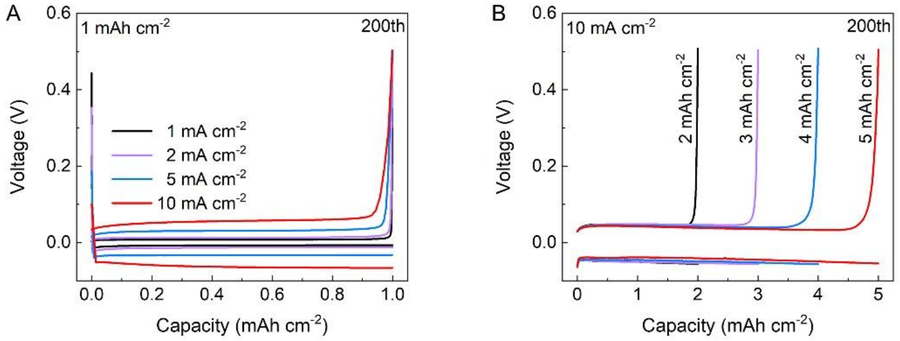

Fig. S29. CE analysis. Voltage-capacity curves of Na//porous Al cell tested at different (A) current densities and (B) areal capacities.

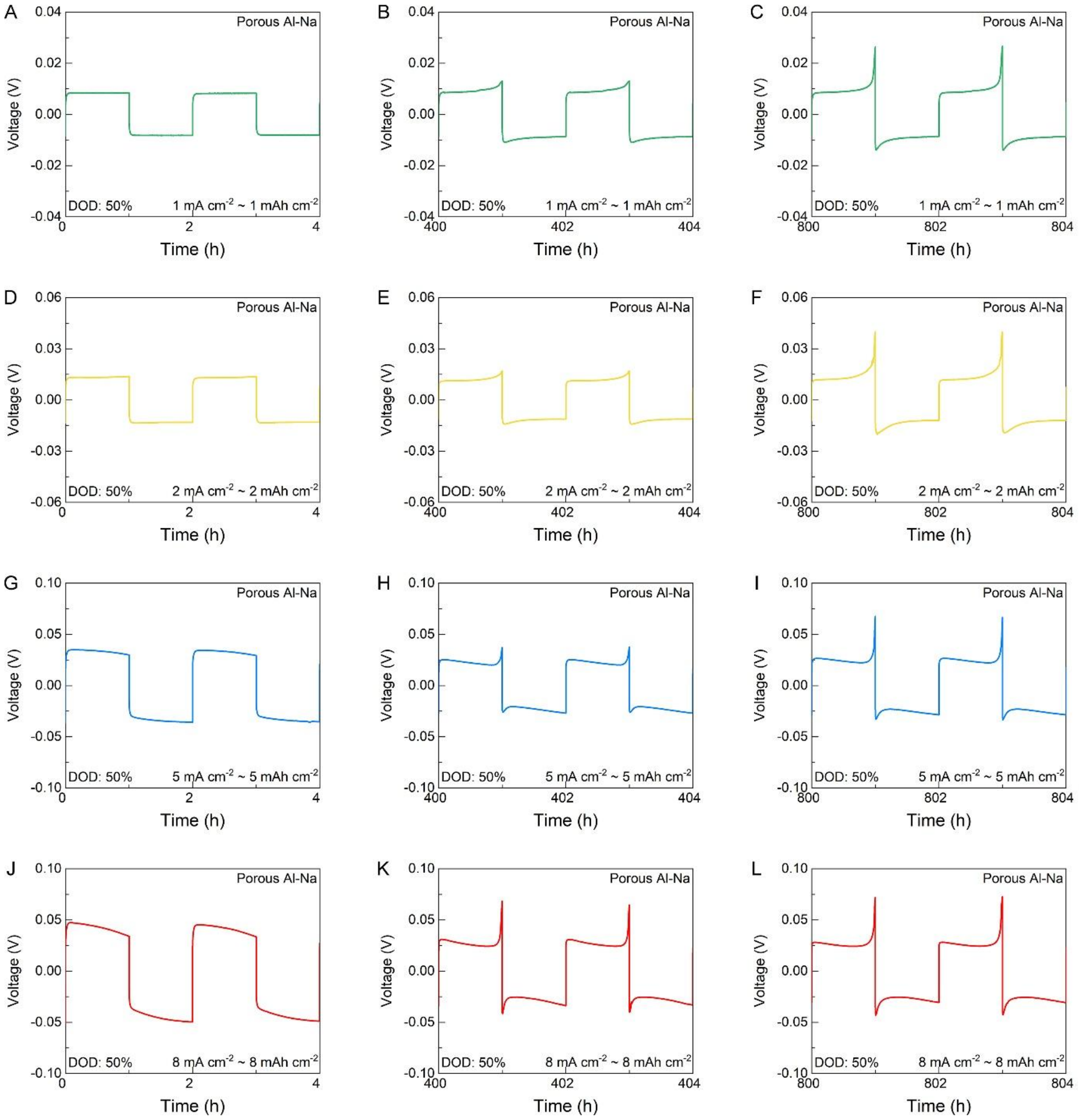

Fig. S30. Cycle analysis. Enlarged voltage profiles of Na//porous Al-Na cell tested at various current densities and areal capacities. (A to C) 1 mA cm^{-2} and 1 mAh cm^{-2}, (D to F) 2 mA cm^{-2} and 2 mAh cm^{-2}, (G to I) 5 mA cm^{-2} and 5 mAh cm^{-2}, (J to L) 8 mA cm^{-2} and 8 mAh cm^{-2}.

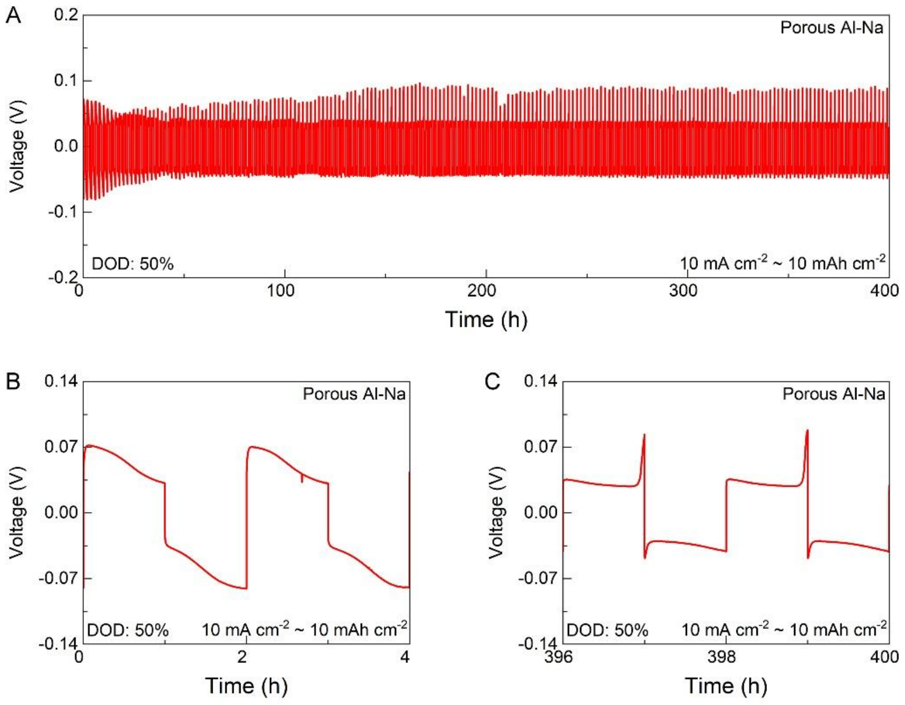

Fig. S31. Cycle analysis. (A) Cycling performance and (B and C) enlarged voltage profiles of Na//porous Al-Na cell tested at 10 mA cm^{-2} and 10 mAh cm^{-2}.

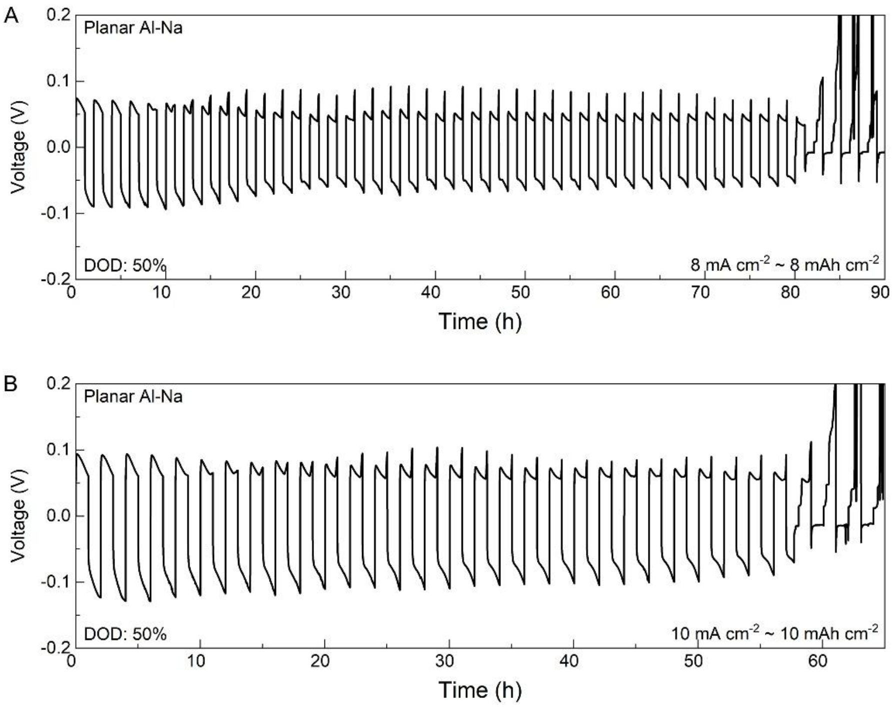

Fig. S32. Cycle analysis. Cycling performance of Na//planar Al-Na cell tested at (A) 8 mA cm^{-2} and 8 mAh cm^{-2}, (B) 10 mA cm^{-2} and 10 mAh cm^{-2}.

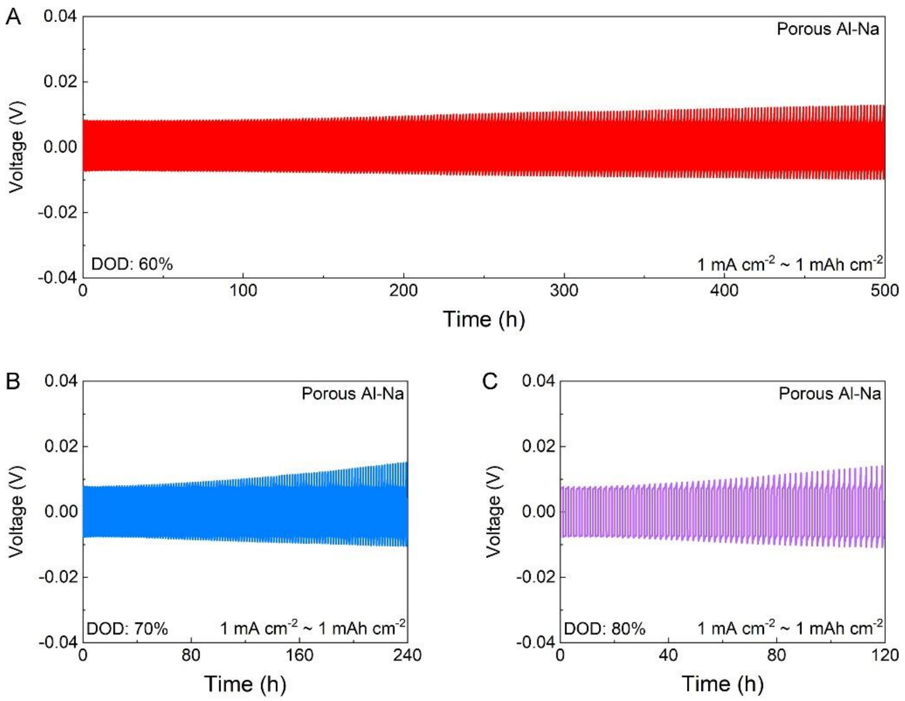

Fig. S33. Cycle analysis. Cycling performance of Na//porous Al-Na cell tested at high DOD values of (A) 60%, (B) 70%, and (C) 80%.

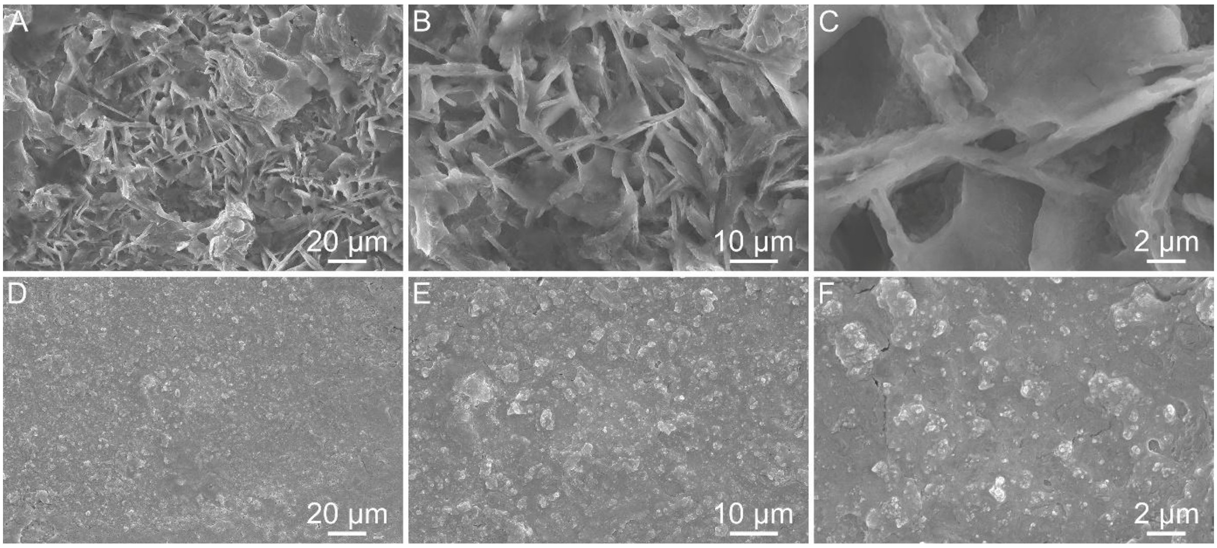

Fig. S34. FESEM measurement. FESEM images of (A to C) planar Al-Na and (D to F) porous Al-Na anodes after cycling.

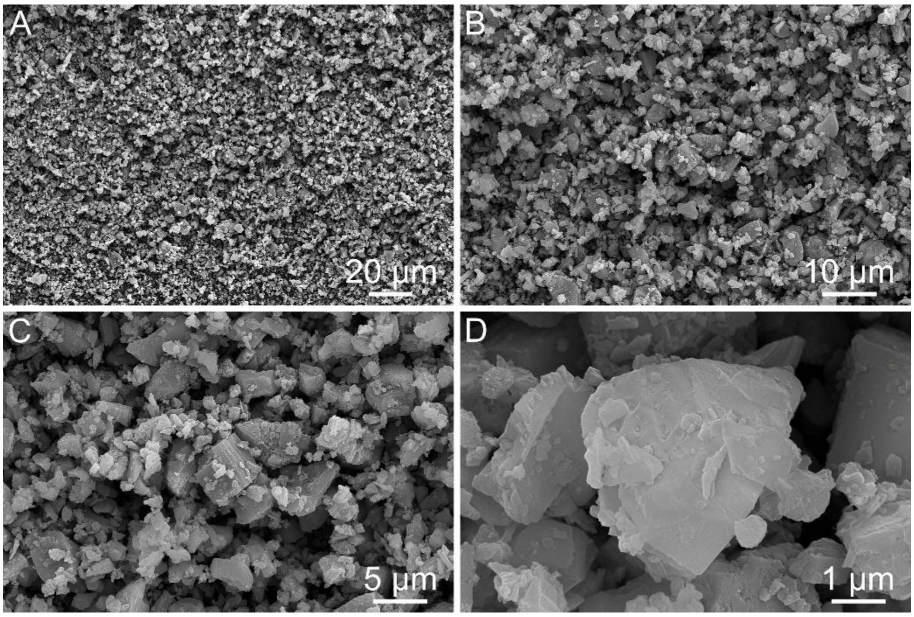

Fig. S35. FESEM measurement. FESEM images of NVOPF.

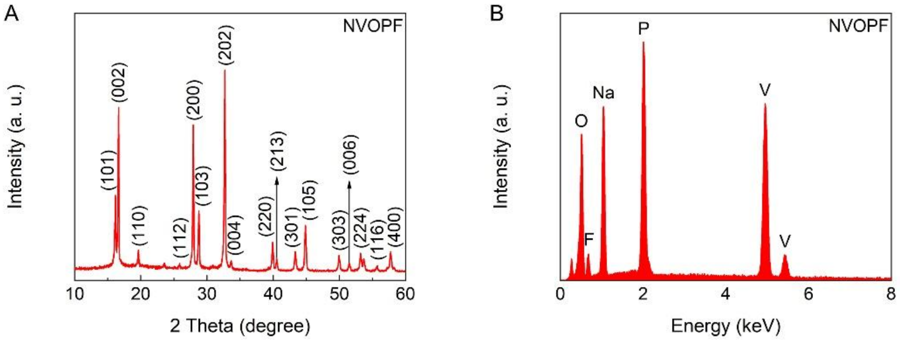

Fig. S36. XRD and EDX measurements. (A) XRD pattern and (B) EDX spectrum of NVOPF.

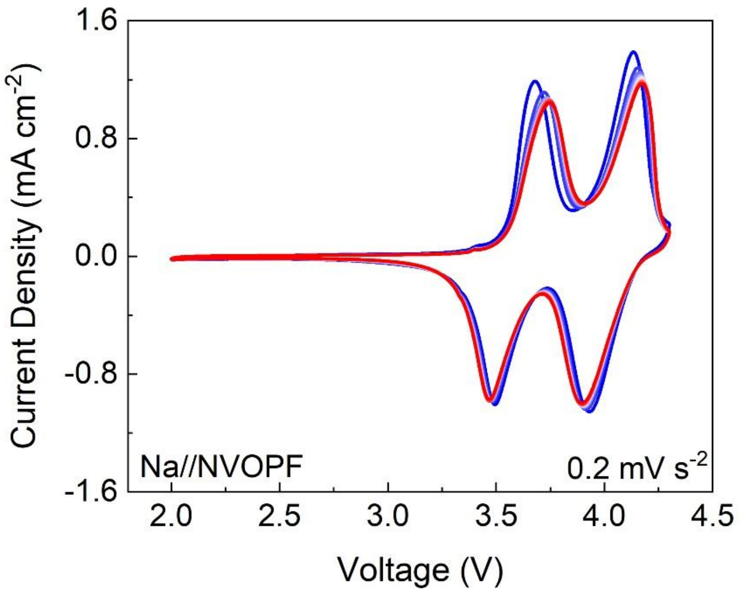

Fig. S37. CV analysis. CV curves of Na//NVOPF cell tested at a scan rate of 0.2 mV s^{-1}.

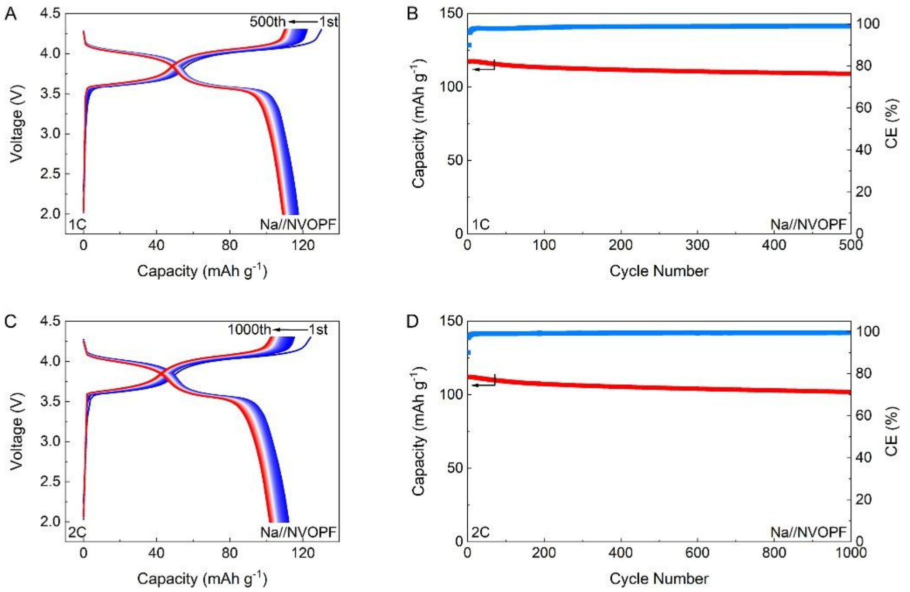

Fig. S38. Cycling analysis. (A and C) Charge-discharge voltage profiles and (B and D) cycling performance of Na//NVOPF cell tested at different current densities of (A and B) 1C and (C and D) 2C.

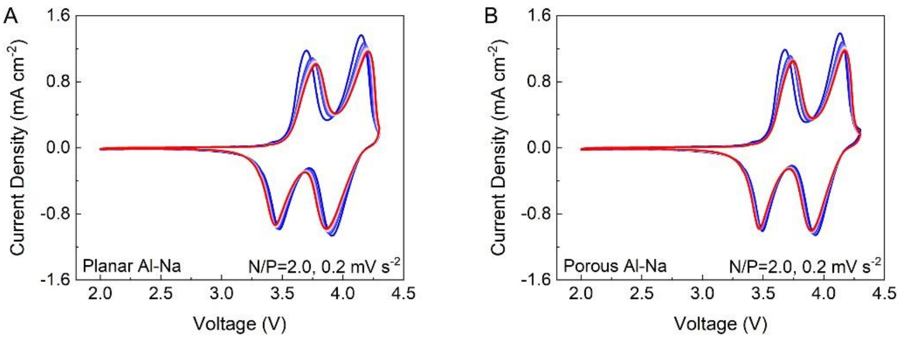

Fig. S39. CV analysis. CV curves of (A) planar Al-Na//NVOPF and (B) porous Al-Na//NVOPF cells (N/P = 2.0) tested at a scan rate of 0.2 mV s^{-1}.

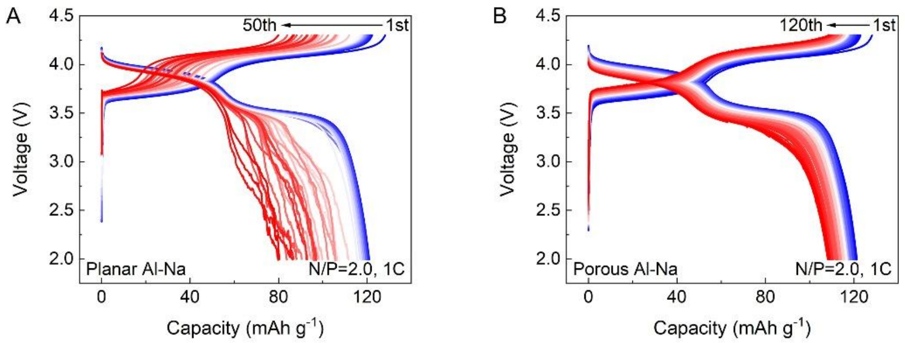

Fig. S40. Cycling analysis. Charge-discharge voltage profiles of (A) planar Al-Na//NVOPF and (B) porous Al-Na//NVOPF cells (N/P = 2.0) tested at a current density of 1C.

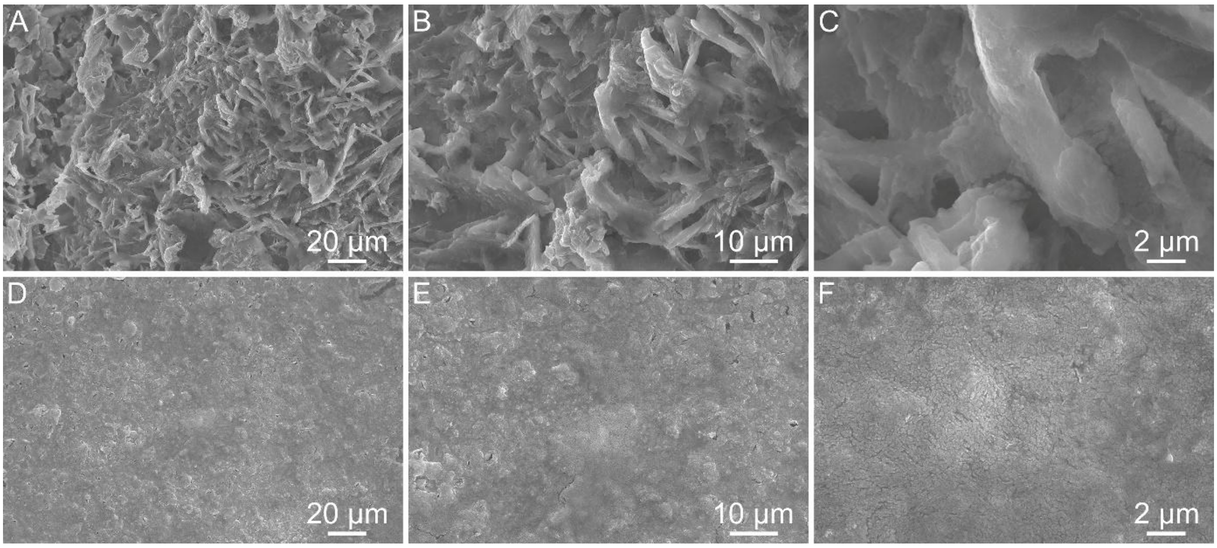

Fig. S41. FESEM measurement. FESEM images of (A to C) planar Al-Na and (D to F) porous Al-Na anodes in full cell after 120 cycles.

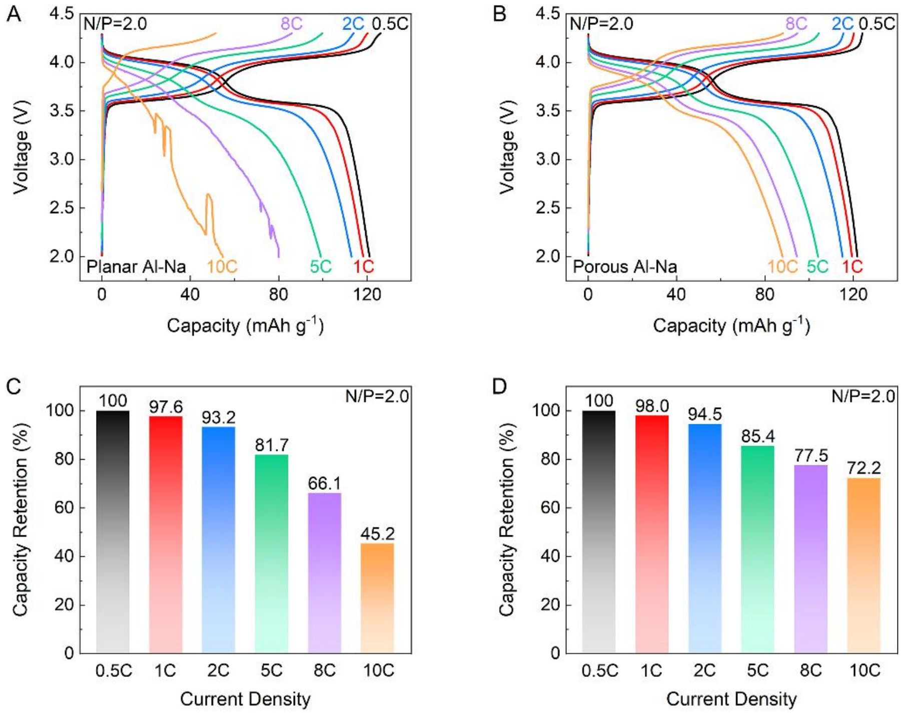

Fig. S42. Rate analysis. (A and B) Charge-discharge voltage profiles and (C and D) capacity retentions of (A and C) planar Al-Na//NVOPF and (B and D) porous Al-Na//NVOPF cells (N/P = 2.0) tested at different current densities from 0.5C to 10C.

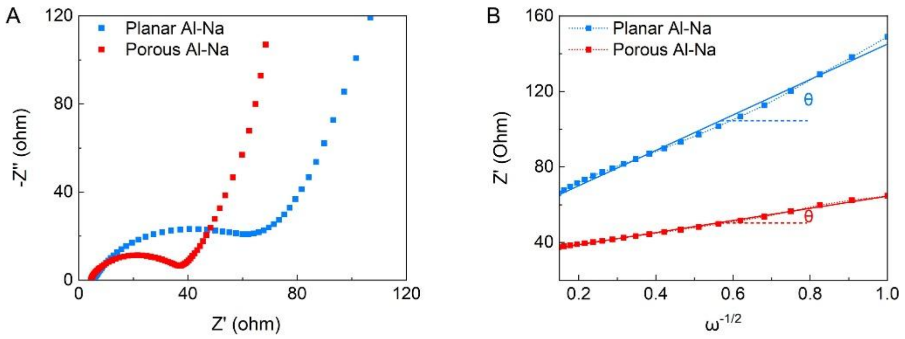

Fig. S43. EIS analysis. (A) Nyquist plots and (B) relationship plots between Z' and $\omega^{-1/2}$ of planar Al-Na//NVOPF and porous Al-Na//NVOPF cells (N/P = 2.0) after 40 cycles.

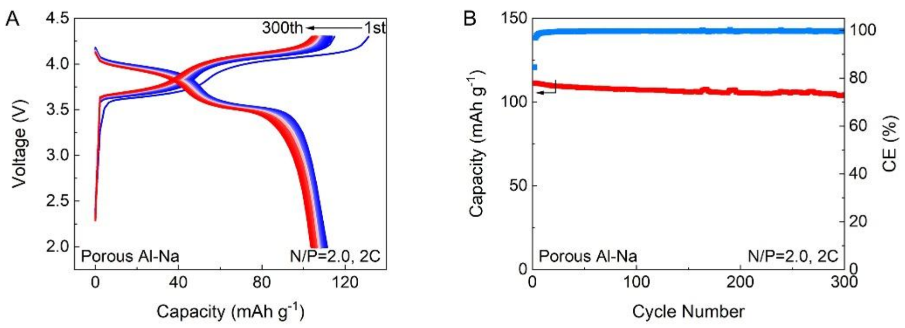

Fig. S44. Cycling analysis. (A) Charge-discharge voltage profiles and (B) cycling performance of porous Al-Na//NVOPF cell (N/P = 2.0) tested at a high current density of 2C.

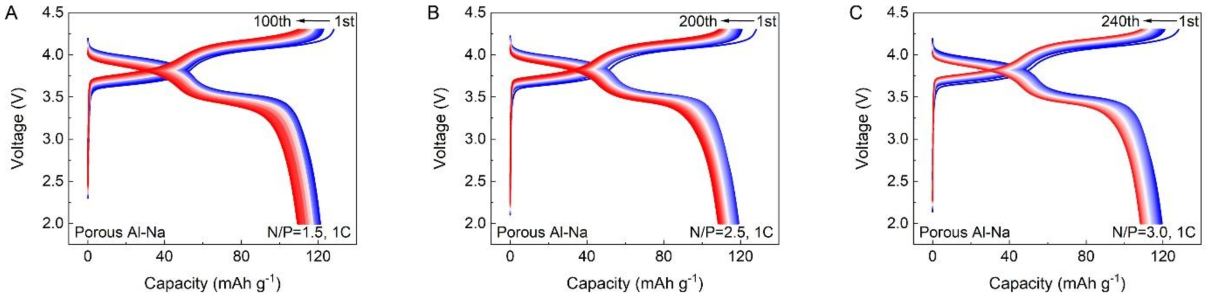

Fig. S45. Cycling analysis. Charge-discharge voltage profiles of porous Al-Na//NVOPF cell with different N/P ratios tested at a current density of 1C. (A) N/P = 1.5, (B) N/P = 2.5, and (C) N/P = 3.0.

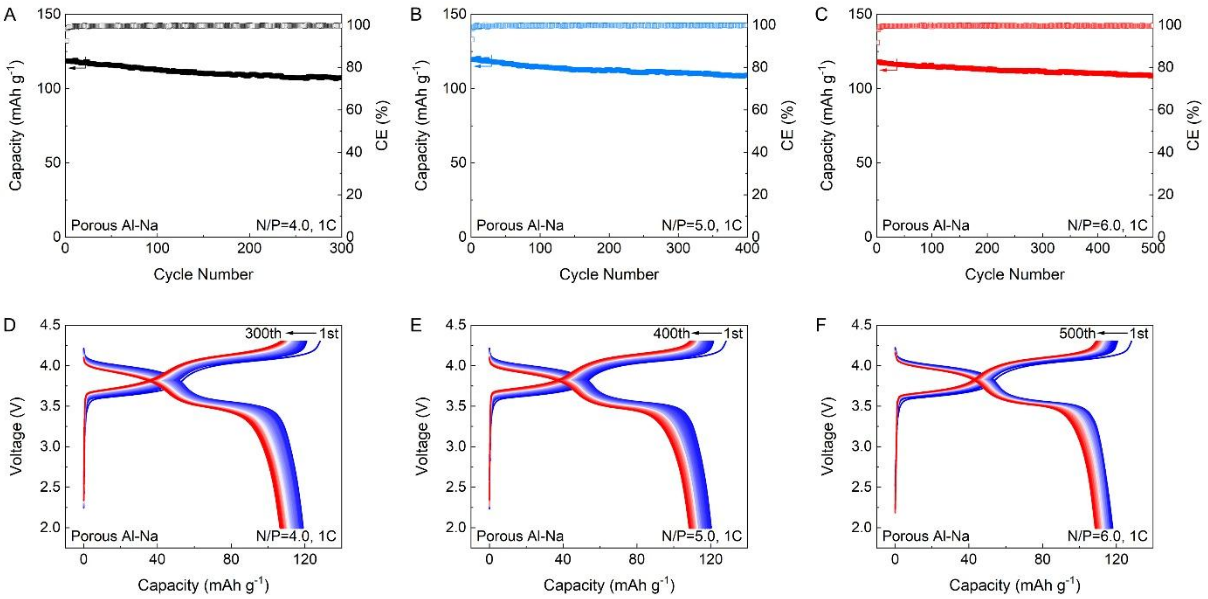

Fig. S46. Cycling analysis. (A to C) Cycling performance and (D to F) charge-discharge voltage profiles of porous Al-Na//NVOPF cell with different N/P ratios tested at a current density of 1C. (A and D) N/P = 4.0, (B and E) N/P = 5.0, and (C and F) N/P = 6.0.

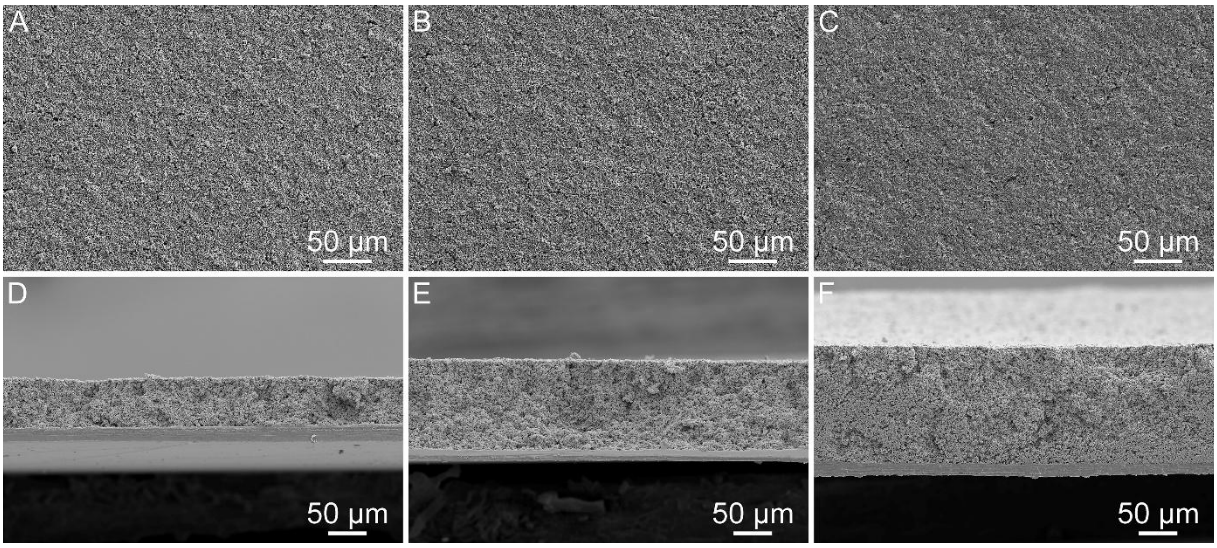

Fig. S47. FESEM measurement. (A to C) Top-view and (D to F) cross-sectional FESEM images of NVOPF cathode with different mass loadings of (A and D) 9 mg cm^{-2}, (B and E) 18 mg cm^{-2}, (C and F) 33 mg cm^{-2}.

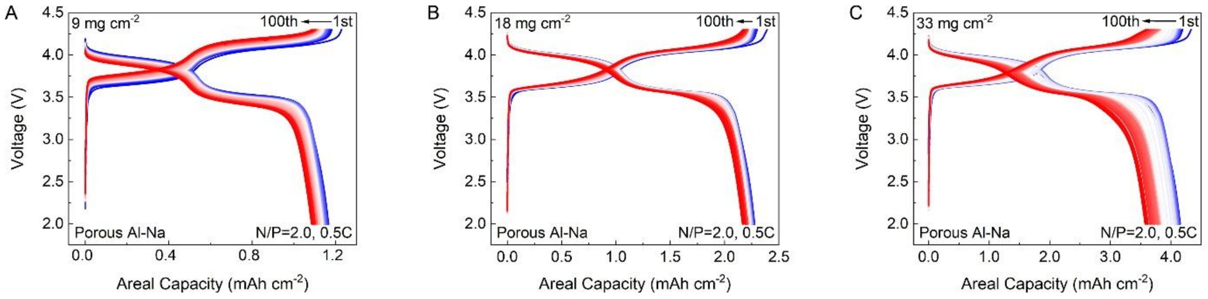

Fig. S48. Cycling analysis. Charge-discharge voltage profiles of porous Al-Na//NVOPF cell with different NVOPF loadings tested at a current density of 0.5C. (A) 9 mg cm^{-2}, (B) 18 mg cm^{-2}, and (C) 33 mg cm^{-2}.

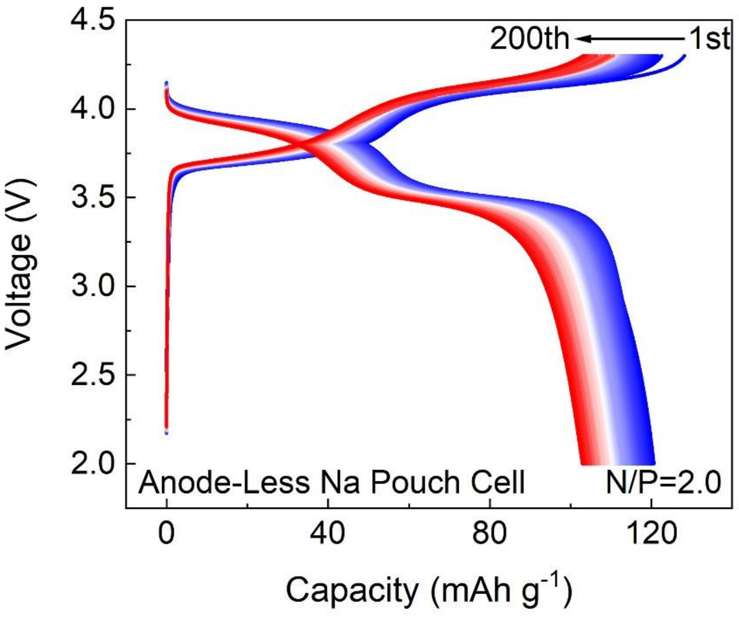

Fig. S49. Cycling analysis. Charge-discharge voltage profiles of porous Al-Na//NVOPF pouch cell (N/P = 2.0) tested at a current density of 0.5C.

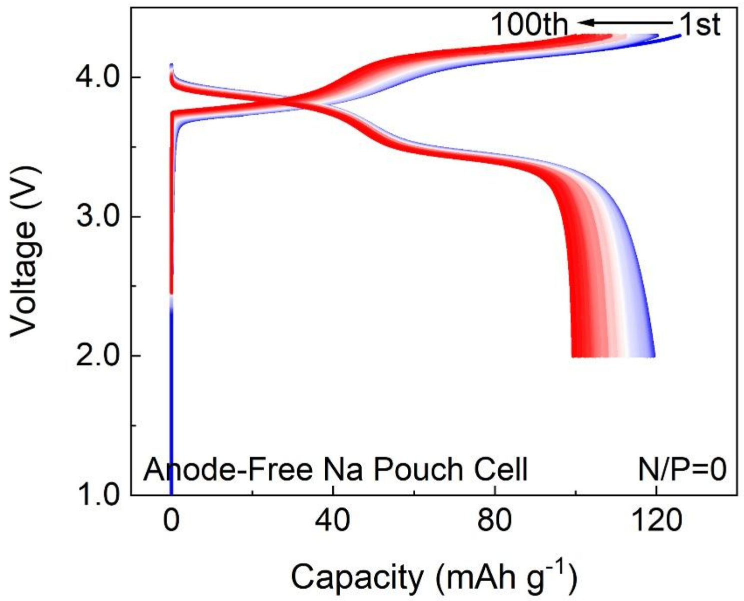

Fig. S50. Cycling analysis. Charge-discharge voltage profiles of anode-free porous Al//NVOPF pouch cell (N/P = 0) tested at a current density of 0.5C.

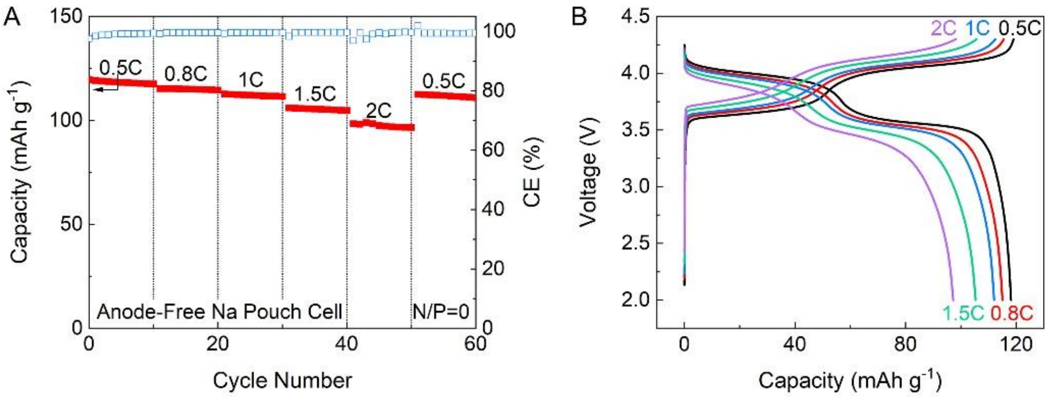

Fig. S51. Rate analysis. (A) Rate capability and (B) corresponding charge-discharge voltage profiles of anode-free porous Al//NVOPF pouch cell (N/P = 0) tested at different current densities from 0.5C to 2C.

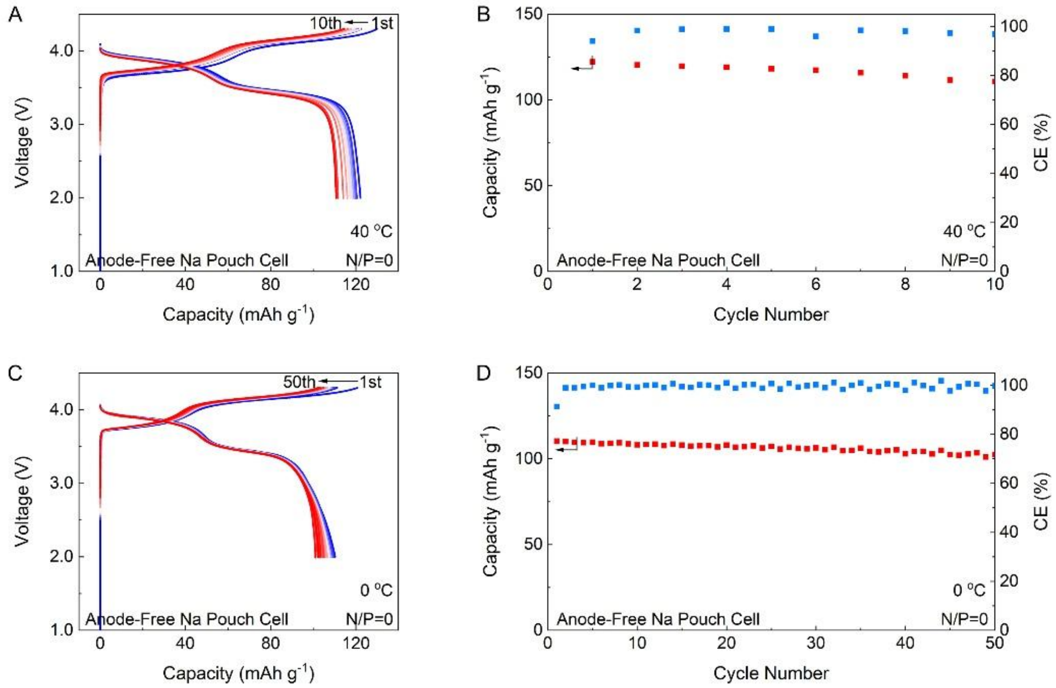

Fig. S52. Cycling analysis. (A and C) Charge-discharge voltage profiles and (B and D) cycling performance of anode-free porous Al//NVOPF pouch cell (N/P = 0) tested at (A and B) 40 ^{o}C and (C and D) 0 ^{o}C.

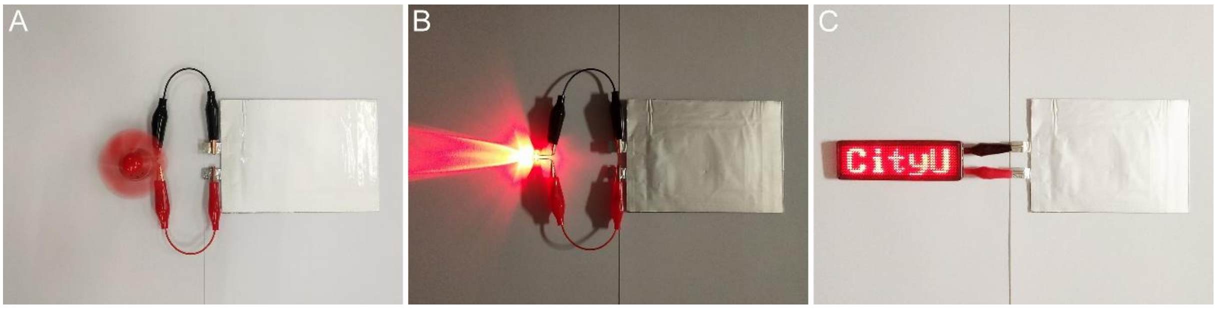

Fig. S53. Digital photograph. Optical images of fully charged anode-free porous Al//NVOPF pouch cells to light up (A) a rotating fan, (B) an LED light, and (C) an LED sign.

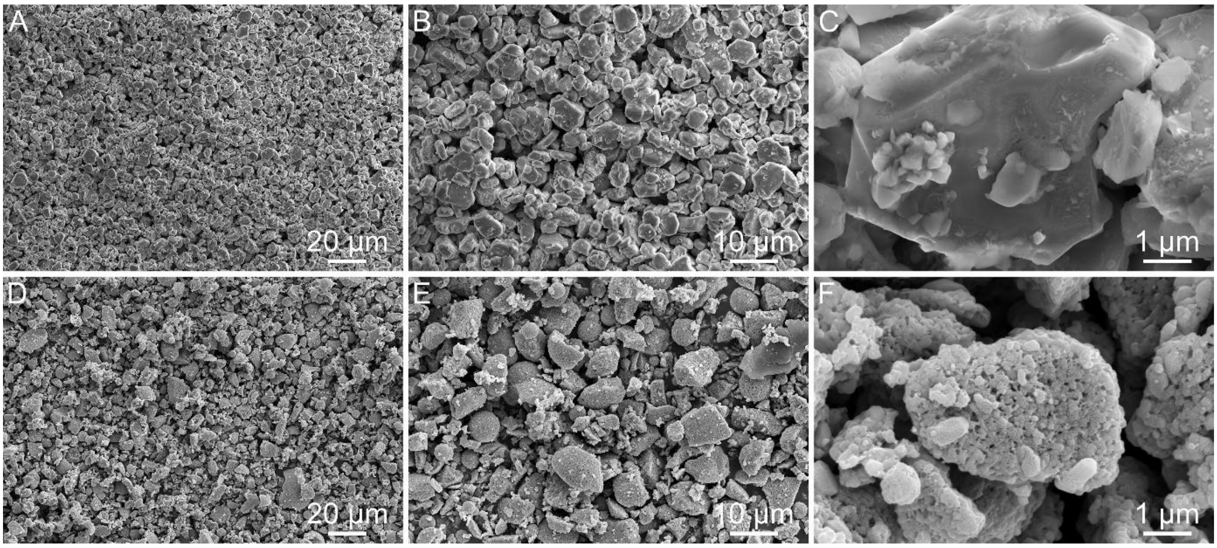

Fig. S54. FESEM measurement. FESEM images of (A to C) NFM and (D to F) NFPP.

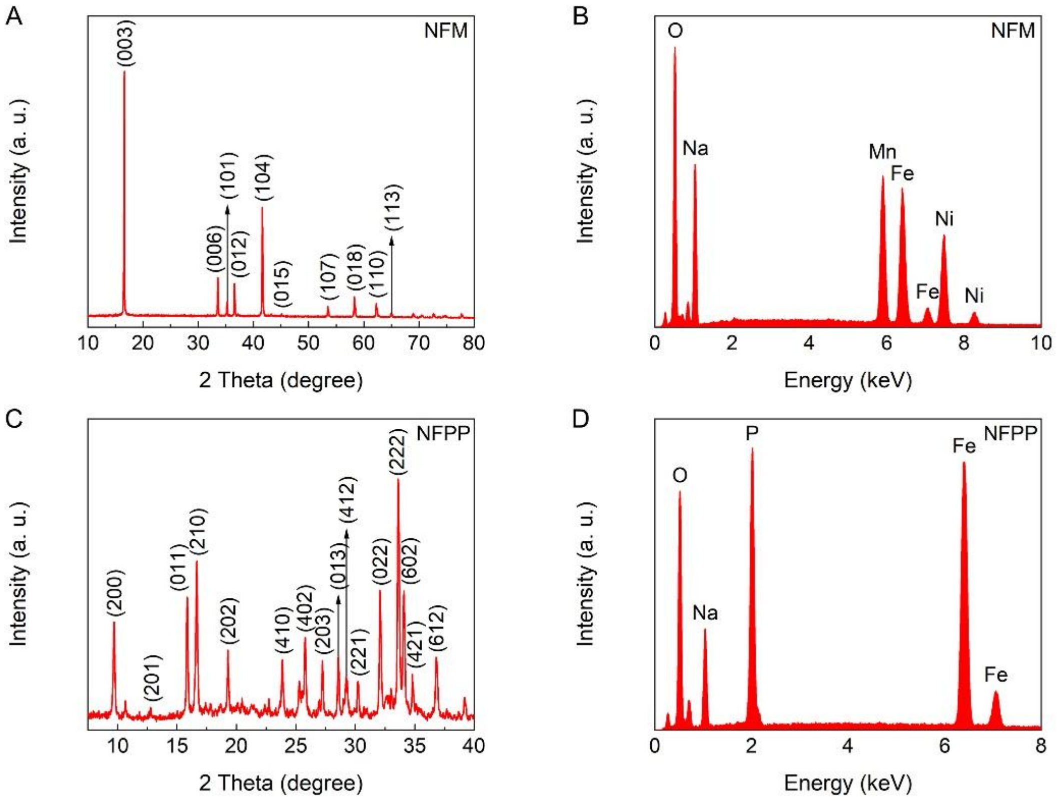

Fig. S55. XRD and EDX measurements. (A and C) XRD patterns and (B and D) EDX spectra of (A and B) NFM and (C and D) NFPP.

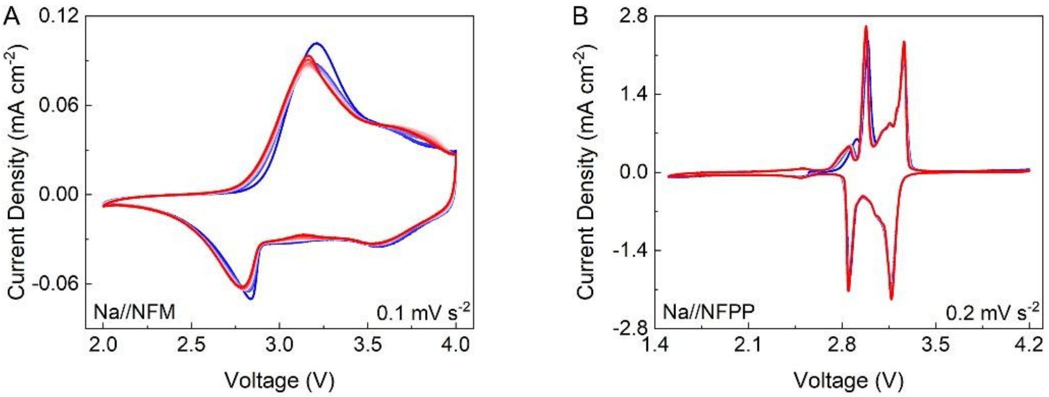

Fig. S56. CV analysis. CV curves of (A) Na//NFM and (B) Na//NFPP cells.

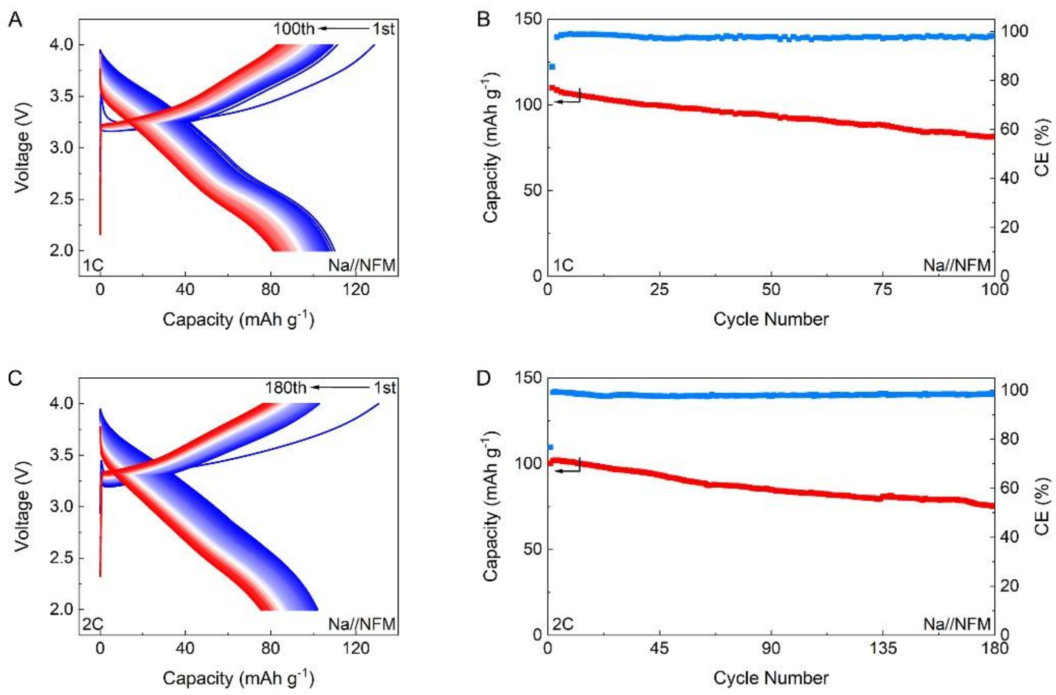

Fig. S57. Cycling analysis. (A and C) Charge-discharge voltage profiles and (B and D) cycling performance of Na//NFM cell tested at different current densities of (A and B) 1C and (C and D) 2C.

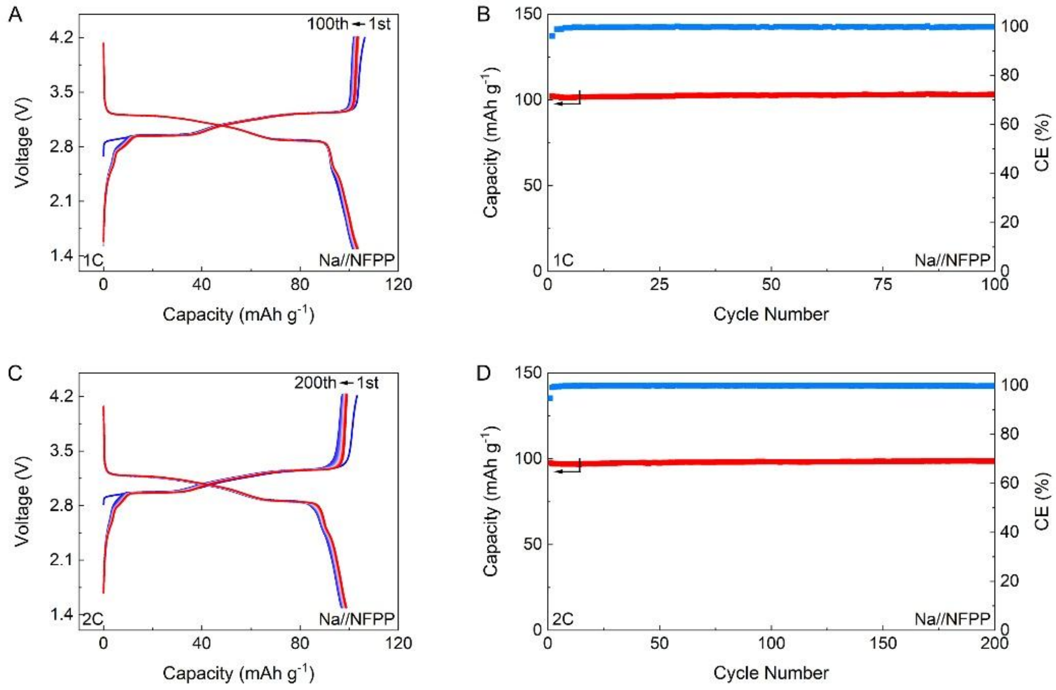

Fig. S58. Cycling analysis. (A and C) Charge-discharge voltage profiles and (B and D) cycling performance of Na//NFPP cell tested at different current densities of (A and B) 1C and (C and D) 2C.

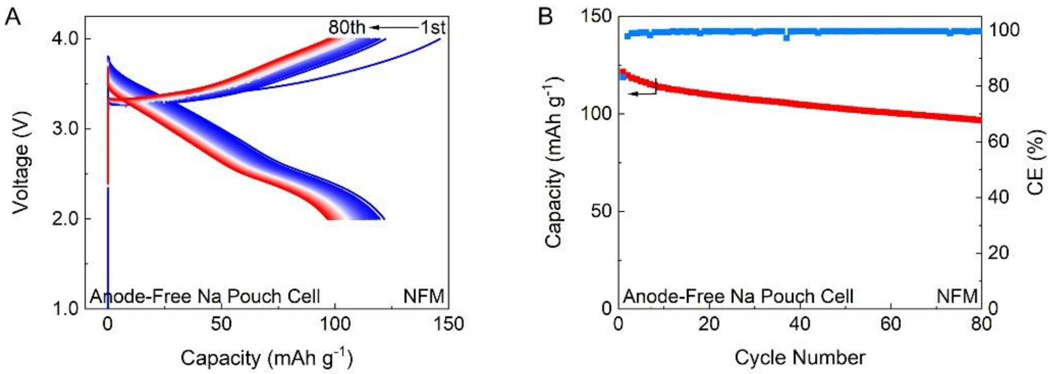

Fig. S59. Cycling analysis. (A) Charge-discharge voltage profiles and (B) cycling performance of anode-free porous Al//NFM pouch cell.

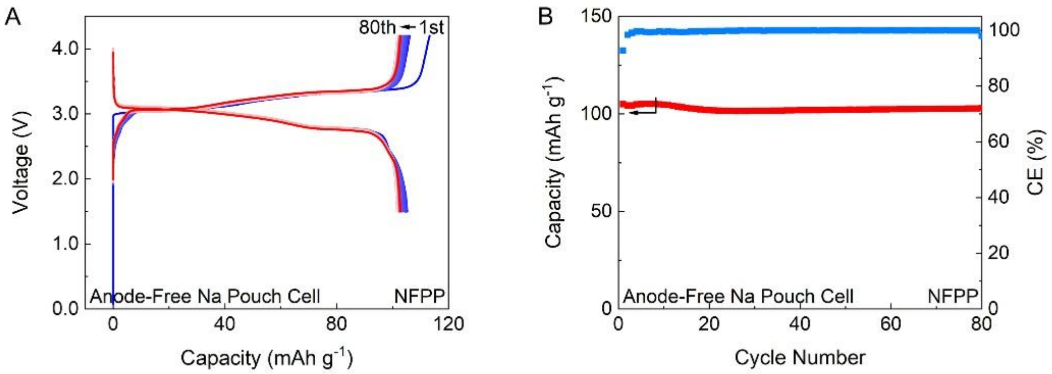

Fig. S60. Cycling analysis. (A) Charge-discharge voltage profiles and (B) cycling performance of anode-free porous Al//NFPP pouch cell.

3. Tables in the Supplementary Materials

Table S1. Comparisons of Na plating/stripping performance of the porous Al tested at different current densities and areal capacities.

Current Density ($mA\ cm^{-2}$)	Areal Capacity ($mAh\ cm^{-2}$)	Cycle Number	Accumulated Capacity ($mAh\ cm^{-2}$)	CE (%)
1	0.5	800	400	~99.91
1	1	1200	1200	~99.98
2	1	2850	2850	~100
5	1	3450	3450	~100
10	1	3900	3900	~99.98
10	2	894	1788	~99.96
10	3	750	2250	~99.95
10	4	534	2136	~99.91
10	5	900	4500	~99.92
10	20	201	4020	~100

Table S2. Comparisons of electrochemical performance for the porous Al and other hosts reported in the previous works.

Electrode	Electrolyte	Current Density (mA cm^{-2})	Areal Capacity (mAh cm^{-2})	Cycles	Accumulated Capacity (mAh cm^{-2})	CE (%)	Ref.
CoP@N/P-CMFs	1M $NaPF_6$ in Diglyme	10	10	330	3300	99.97	(*19*)
SnNCNFs	1M $NaPF_6$ in Diglyme	3	3	2000	6000	99.96	(*45*)
Al-Cu@C	1M $NaPF_6$ in Diglyme	0.5	1	60	60	97.5	(*37*)
PC-CFe	1M $NaPF_6$ in Diglyme	10	10	500	5000	99.6	(*39*)
O-CCF	1M $NaPF_6$ in Diglyme	5	10	1000	10000	99.6	(*40*)
OCF	0.01 M NaTFSI + 1M $NaSO_3CF_3$ in Diglyme	10	1	2500	2500	99.83	(*46*)
FCTF	1M $NaPF_6$ in Diglyme	2	1	400	400	99.6	(*47*)
a-CNTs	1M $NaSO_3CF_3$ in Diglyme	3	1	1000	1000	99.8	(*48*)
MgF_2@RGO	1M $NaClO_4$ in EC/DEC + 5% FEC	0.5	0.5	325	162.5	~96	(*49*)
NOCS	0.9 M $NaPF_6$ + 0.1 M $NaBF_4$ in Diglyme	2	2	1000	2000	99.97	(*50*)
2D Sn/C	1M $NaSO_3CF_3$ in Diglyme	2	2	970	1940	99.85	(*51*)
CT-Sn(II)@Ti_3C_2	1M $NaPF_6$ in Diglyme	10	3	100	300	98.5	(*52*)
At-Sn@HCN	1M $NaPF_6$ in Diglyme	2	1	1000	1000	99.93	(*4*)
NiSb-CC	1M $NaClO_4$ in EC/DEC + 5% FEC	-	3.5	150	525	99.74	(*53*)
NSCNT	1M $NaSO_3CF_3$ in Diglyme	1	1	400	400	99.82	(*54*)
MgF_2@NCH NFs	1M $NaPF_6$ in Diglyme	5	1	2000	2000	99.9	(*55*)
Cu_3P@Cu	1M $NaPF_6$ in Diglyme	4	4	250	1000	99.12	(*18*)
N-CSs/Cu	1M $NaPF_6$ in Diglyme	2	2	900	1800	99.99	(*56*)
Cu	1M $NaPF_6$ in Diglyme + SiO_2	1	2	500	1000	-	(*57*)
Cu	2.0 M $NaPF_6$ in DEE	1	1	150	150	99.89	(*5*)

Electrode	Electrolyte	Current Density (mA cm^{-2})	Areal Capacity (mAh cm^{-2})	Cycles	Accumulated Capacity (mAh cm^{-2})	CE (%)	Ref.
Cu@Sn	0.01M NaTFSI + 1M NaOTf in Diglyme	2	1	2000	2000	99.9	(*58*)
Cu@Sb	0.01M NaTFSI + 1M NaOTf in Diglyme	2	1	1600	1600	99.9	(*58*)
Cu@Au	1M $NaSO_3CF_3$ in Diglyme	2	2	300	600	99.8	(*59*)
Bi-NAs@Cu	1M $NaPF_6$ in Diglyme	1	1	1000	1000	99.8	(*60*)
KJB@Cu	1M $NaPF_6$ in Diglyme	0.5	1	150	150	99.8	(*27*)
Al	Na-SSZE	1	1	1000	1000	99.84	(*25*)
Porous Al	1M $NaPF_6$ in Diglyme	1	0.5	1000	500	99.8	(*38*)
Carbon/Al	1M $NaPF_6$ in Diglyme	0.5	0.25	1000	250	99.8	(*61*)
3D Zn@Al	1 M $NaPF_6$ in DME	2	2	500	1000	99.5	(*62*)
p-Al@C	0.6 M NaOTF + 0.4 M $NaBF_4$ in Diglyme	0.5	0.5	100	50	99.88	(*17*)
BTO/C-Al	$Na_3Zr_2Si_2PO_{12}$	0.1	1	165	165	98.2	(*24*)
NbMoTaWV @Al	1 M $NaPF_6$ in DME	2	2	1000	2000	99.5	(*26*)
Sb_2MoO_6	1M $NaPF_6$ in Diglyme	5	4	500	2000	95.2	(*63*)
3D-NVP	1M $NaClO_4$ in PC + 5% FEC	1	1	190	190	98	(*64*)
L700	1M $NaSO_3CF_3$ in Diglyme	1	2	300	600	99.6	(*65*)
Porous Al	1M $NaPF_6$ in Diglyme	10	20	225	4500	99.99	This Work

Table S3. Comparisons of cycling performance of the porous Al-Na anodes with different current densities and areal capacities.

Current Density (mA cm^{-2})	Areal Capacity (mAh cm^{-2})	Depth-of-Discharge (%)	Cycle Number	Time (h)	Accumulated Capacity (mAh cm^{-2})
1	1	50	400	800	400
2	2	50	400	800	800
5	5	50	400	800	2000
8	8	50	400	800	3200
10	10	50	200	400	2000

Table S4. Comparisons of electrochemical performance for the porous Al-Na and other composite Na anodes reported in the previous works.

Electrode	Electrolyte	Current Density (mA cm^{-2})	Areal Capacity (mAh cm^{-2})	Depth-of-Discharge (%)	Accumulated Capacity (mAh cm^{-2})	Life (h)	Ref.
FCTF-Na	1M $NaPF_6$ in Diglyme	2	2	100	140	140	(*47*)
CoP@N/P-CMFs-Na	1M $NaPF_6$ in Diglyme	8	8	50	3200	800	(*19*)
SnNCNFs	1M $NaPF_6$ in Diglyme	10	10	86	3500	700	(*45*)
N-CSs-Na	1M $NaPF_6$ in Diglyme	2	2	-	1500	1500	(*56*)
NST-Na	1M $NaPF_6$ in Diglyme	2	10	60	1500	1500	(*66*)
PC-CFe-Na	1M $NaPF_6$ in Diglyme	5	5	-	4125	1650	(*39*)
O-CCF-Na	1M $NaPF_6$ in Diglyme	50	1	16.67	10000	400	(*40*)
OCF-Na	0.01 M NaTFSI + 1.0 M CF_3NaO_3S in Diglyme	5	5	-	5000	2000	(*46*)
a-CNTs-Na	1M $NaSO_3CF_3$ in Diglyme	5	8	-	750	300	(*48*)
MgF_2@RGO-Na	1M $NaClO_4$ in EC/DEC + 5% FEC	0.5	0.5	10	400	1600	(*49*)
NOCS-Na	0.9 M $NaPF_6$ + 0.1 M $NaBF_4$ in Diglyme	0.5	1	50	250	1000	(*50*)
2D Sn/C-Na	1M $NaSO_3CF_3$ in Diglyme	3	3	50	150	100	(*51*)
CT-Sn(II)@Ti_3C_2-Na	1M $NaPF_6$ in Diglyme	5	3	60	600	240	(*52*)
At-Sn@HCN-Na	1M $NaPF_6$ in Diglyme	4	8	80	10000	5000	(*4*)
NiSb-CC-Na	1M $NaClO_4$ in EC/DEC + 5% FEC	1	10	47	500	1000	(*53*)
NSCNT-Na	1M $NaSO_3CF_3$ in Diglyme	1	1	-	250	500	(*54*)
MgF_2@NCHNFs-Na	1M $NaPF_6$ in Diglyme	5	2	16.7	9000	3600	(*55*)
Cu_3P@Cu-Na	1M $NaPF_6$ in Diglyme	2	2	-	2000	2000	(*18*)
Bi-NAs@Cu	1M $NaPF_6$ in Diglyme	1	3	75	600	1200	(*60*)
Porous Al-Na	1M $NaPF_6$ in Diglyme	0.5	0.5	25	250	1000	(*38*)
3D Zn@Al-Na	1 M $NaPF_6$ in DME	2	1	50	1500	1500	(*62*)
F-A-Al-Na	Na[FSA] in	0.5	0.5	25	150	600	(*31*)

Electrode	Electrolyte	Current Density (mA cm^{-2})	Areal Capacity (mAh cm^{-2})	Depth-of-Discharge (%)	Accumulated Capacity (mAh cm^{-2})	Life (h)	Ref.
	[C2C1im][FSA]						
BTO/C-Al-Na	$Na_3Zr_2Si_2PO_{12}$	0.5	0.5	-	125	500	(*24*)
NbMoTaWV @Al	1 M $NaPF_6$ in DME	2	1	50	1000	1000	(*26*)
HCOONa-Na	1M $NaPF_6$ in Diglyme	2	1	-	2200	2200	(*67*)
Sb_2MoO_6-Na	1M $NaPF_6$ in Diglyme	10	8	-	960	192	(*63*)
3D-NVP-Na	1M $NaClO_4$ in PC + 5% FEC	1	2	20	200	400	(*64*)
L700-Na	1M $NaSO_3CF_3$ in Diglyme	1	0.5	-	500	1000	(*65*)
Porous Al-Na	1M $NaPF_6$ in Diglyme	10	10	50	3500	700	This Work

Table S5. Comparisons of electrochemical performance of the anode-less porous Al-Na//NVOPF cell with previously reported works.

Anode	Cathode	Mass Loading (mg cm^{-2})	N/P Ratio	Voltage (V)	Current Density (mA g^{-1})	Capacity (mAh g^{-1})	Cycle Number	Capacity Retention (%)	Ref.
NOCS-Na	NVP	11.4	1.5	2.0-3.8	500	100	800	96.2	(*50*)
Bi-NAs@Cu-Na	NTP	6.0	1.0	1.5-2.5	133	95.18	267	93.22	(*60*)
KJB@Cu-Na	NVP	-	-	2.5-3.8	60		150	94.16	(*27*)
FCTF-Na	NVP	6.4	1.5	2.5-3.8	236	108.9	300	94.7	(*47*)
SnNCNF-Na	S@pPAN	10	1.5	0.6-2.8	750	-	80	81	(*45*)
N-CSs	NNMO	4	-	2.5-3.9	-	-	200	86.3	(*56*)
Porous Al-Na	NVOPF	33	2.0	2.0-4.3	65	108.0	100	86.2	This Work

Table S6. Comparisons of electrochemical performance of the anode-free porous Al//NVOPF cell with previously reported works.

Anodic Current Collector	Cathode	Mass Loading (mg cm^{-2})	Voltage (V)	Current Density (mA g^{-1})	Capacity (mAh g^{-1})	Cycle Number	Capacity Retention (%)	Ref.
CoP@N/P-CMFs	NVP	18	2.0-3.0	117	82.7	120	87.38	(*19*)
NOCS	NVP	12.3	2.0-3.8	200	81.4	350	79.0	(*50*)
FCTF	NVP	7.4	2.5-3.8	240	-	400	56	(*47*)
MgF_2@NCHNFs	NVP	8	2.5-3.8	236	-	50	91.2	(*55*)
SnNCNF	NVP	10	2.6-3.8	234	89.3	80	89	(*45*)
PC-CFe	NVP	10	2.6-3.8	1 mA cm^{-2}	103	100	97	(*39*)
O-CCF	NVP	11	2.5-4.0	1 mA cm^{-2}	103.7	100	96	(*40*)
Cu	NFM	4.5	2.0-3.8	70	-	100	82.3	(*5*)
Cu	NVP	4	2.6-3.8	0.5 mA cm^{-2}	71.88	100	75	(*57*)
Cu@Au	Na-FeS_2	-	0.8-3.0	-	140	50	-	(*59*)
Cu_3P@Cu	NVP	13.6	2.5-3.5	60	76.1	75	-	(*18*)
SF-Cu	NVP	10	2.8-3.7	58.5	86.3	400	88	(*67*)
C@Al	NVPOF	7.1	2.0-4.25	0.49 mA cm^{-2}	97.9	370	89.2	(*25*)
C@Al	NNCFM	8.52	2.0-4.0	30	63.9	250	-	(*68*)
GC@Al	NCNFM	15.12	2.0-3.8	63.5	-	260	84	(*34*)
F-A-Al	NVP	11.3	2.4-3.6	35.1	-	50	46.1	(*31*)
3D Zn@Al	NVP	1.8	2.5-3.8	59	-	100	98.8	(*62*)
BTO/C-Al	NVP	-	2.5-3.8	0.1 mA cm^{-2}	104.1	300	95.5	(*24*)
NbMoTaWV@Al	NVP	1.8	2.5-3.8	-	-	300	96.5	(*26*)
NST	NVP	2.5	3.2-3.6	118	73.15	100	77	(*66*)
Al-Cu@C	NVP/C	3.5-4.0	1.5-3.8	120	-	50	-	(*37*)
Porous Al	Na-TiS_2	-	1.3-2.6	0.1 mA cm^{-2}	~160	200	~64	(*38*)
Carbon/Al	$Na_{1.5}FeS_2$	5	0.8-3.0	0.125 mA cm^{-2}	335	40	-	(*61*)
N-CPs	NNMO	4	2.5-3.8	50	-	200	86.3	(*56*)
Porous Al	NVOPF	6.8	2.0-4.3	65	99.9	100	84.9	This Work
